# Differential Regulation and Production of Secondary Metabolites among Isolates of the Fungal Wheat Pathogen Zymoseptoria tritici

**DOI:** 10.1128/aem.02296-21

**Published:** 2022-03-22

**Authors:** M. Amine Hassani, Ernest Oppong-Danquah, Alice Feurtey, Deniz Tasdemir, Eva H. Stukenbrock

**Affiliations:** a Environmental Genomics, Christian-Albrechts University of Kiel, Kiel and Max Planck Institute for Evolutionary Biology, Plön, Germany; b GEOMAR Centre for Marine Biotechnology (GEOMAR‐Biotech), Research Unit Marine Natural Products Chemistry, GEOMAR Helmholtz Centre for Ocean Research Kiel, Kiel, Germany; c Faculty of Mathematics and Natural Science, University of Kiel, Kiel, Germany; Nanjing Agricultural University

**Keywords:** genome evolution, gene clusters, gene regulation, chemodiversity, histone modifications, pathogenicity, metabolomics, feature-based molecular network

## Abstract

The genome of the wheat-pathogenic fungus Zymoseptoria tritici represents extensive presence-absence variation in gene content. Here, we addressed variation in biosynthetic gene cluster (BGC) content and biochemical profiles among three isolates. We analyzed secondary metabolite properties based on genome, transcriptome, and metabolome data. The isolates represent highly distinct genome architecture but harbor similar repertoires of BGCs. Expression profiles for most BGCs show comparable patterns of regulation among the isolates, suggesting a conserved biochemical infection program. For all three isolates, we observed a strong upregulation of a putative abscisic acid (ABA) gene cluster during biotrophic host colonization, indicating that *Z. tritici* interferes with host defenses by the biosynthesis of this phytohormone. Further, during *in vitro* growth, the isolates show similar metabolomes congruent with the predicted BGC content. We assessed if secondary metabolite production is regulated by histone methylation using a mutant impaired in formation of facultative heterochromatin (H3K27me3). In contrast to other ascomycete fungi, chromatin modifications play a less prominent role in regulation of secondary metabolites. In summary, we show that *Z. tritici* has a conserved program of secondary metabolite production, contrasting with the immense variation in effector expression, and some of these metabolites might play a key role during host colonization.

**IMPORTANCE** Zymoseptoria tritici is one of the most devastating pathogens of wheat. So far the molecular determinants of virulence and their regulation are poorly understood. Previous studies have focused on proteinaceous virulence factors and their extensive diversity. In this study, we focus on secondary metabolites produced by *Z. tritici*. Using a comparative framework, we characterize core and noncore metabolites produced by *Z. tritici* by combining genome, transcriptome, and metabolome data sets. Our findings indicate highly conserved biochemical profiles with contrasting genetic and phenotypic diversity of the field isolates investigated here. This discovery has relevance for future crop protection strategies.

## INTRODUCTION

Fungal genomics has revealed a large and untapped diversity of biosynthetic gene clusters (BGCs) encoding secondary metabolites (1). These metabolites have a broad spectrum of biological functions and play an essential role as determinants of fungal life style and niche specialization. Most of the secondary metabolites produced by fungi derive from nonribosomal peptides, polyketides, or mixed polyketide-nonribosomal peptides or other biosynthesis pathways (such as terpenes) (2). Genes involved in the biosynthesis of secondary metabolites are often physically linked in gene clusters, allowing coregulation of gene expression and metabolite production (3). Commonly, BGCs encode the enzymes that are responsible for biosynthesis of the metabolite backbone, including nonribosomal peptide synthetases (NRPS), polyketide synthases (PKS), and fusions of PKS and NRPS (PKS-NRPS/NRPS-PKS hybrids) as well as other enzymes involved in further modifications of the metabolite backbone. Moreover, certain BGCs have genes involved in metabolite transport and/or genes conferring resistance to the activity of the metabolite.

Plant-associated fungi produce a multitude of secondary metabolites to facilitate host invasion, manipulate host defenses, interact with microorganisms, and sequester essential nutrients in plant tissues (4). To this end, it has been demonstrated that fungal pathogens can produce and secrete iron-chelating metabolites, the so-called siderophores, to acquire and sequester iron from plant tissues and the environment (5). Moreover, many fungi, including plant pathogens, produce plant hormones such as gibberellins, abscisic acid (ABA), auxin, and cytokinin, which may interfere with the hormone balance of plants that are colonized by these fungi. However, the regulation and relevance of these secondary metabolites in plant-fungus interactions is still little known (6–8).

Genome data have provided detailed insights into the diversity in BGCs among closely related plant-associated fungi (e.g., see references 9–11); however, to which extent and how this diversity is translated into chemical diversity has been addressed in only a few species. Genome sequencing across diverse taxa has shown that the organization of BGCs in fungal genomes can be highly variable between closely related species and in some cases even between different strains of the same species with various numbers of gene clusters and gene content. Members of the fungal family Clavicipitaceae include plant-associated species with diverse lifestyles, ranging from mutualistic symbionts and endophytes to pathogens. This group of fungi exhibits an exceptional chemotypic diversity originating from diverse architecture of gene clusters responsible for secondary metabolite production (9). Comparative genome analyses in other groups of ascomycete plant pathogens have likewise revealed structural variation associated with BGCs, for example, in the gene clusters responsible for synthesis of the phytotoxins botrydial and botcinic acids in *Botrytis* species (12) and in gene clusters responsible for the synthesis of distinct secondary metabolites, including the mycotoxin fucosarin, in isolates of Fusarium fujikuroi (13). This variation in BGC content and composition may reflect the effect of diversifying selection acting on genes involved in antagonistic plant-pathogen interactions.

Fungi accommodate different environmental and ecological conditions by regulating and optimizing the production of secondary metabolites. This regulation can occur at different levels from individual genes and clusters to global regulation of BGCs and involve complex networks of regulatory components (2). In several fungal pathogen taxa, including species of Fusarium and *Epichloë*, chromatin modifications such as histone methylation or acetylation have been shown to play an important role as global regulators of BGCs as well as regulators of specific gene clusters (14–17). In the fungal pathogen Fusarium graminearum, secondary metabolism is largely regulated by the silencing histone mark H3K27me3. This was demonstrated by deletion of the histone methyltransferase *kmt6*, responsible for the heterochromatin-associated trimethylation of lysine 27 on histone H3 (H3K27me3). The mutant exhibited constitutive expression of secondary metabolite clusters along the genome, severely impacting fungal fitness (14). The cluster organization of biosynthesis genes was proposed favor a chromatin-based regulation of secondary metabolite production, as it allows transcriptional activation in narrowly restricted regions (2).

We have recently described extensive variation in genome composition among closely related species of grass pathogens in the genus *Zymoseptoria* (18). This genus includes the important wheat pathogen *Z. tritici*, which has devastating impacts on wheat production worldwide. Population genomic sequencing of *Z. tritici* has revealed extensive genetic variation along the genome (19), and it is hypothesized that this variation allows the fungus to rapidly adapt to changes in the environment, including new host resistances and fungicide treatments applied in wheat fields. *In silico* predictions of gene clusters have demonstrated how BGCs are enriched in the more rapidly evolving subtelomeric regions of the genome of *Z. tritici*, which may facilitate genetic diversification of the BGCs (20). *Z. tritici* is also characterized by an exceptionally high variability in growth morphology, for example, some isolates being highly melanized and others nonmelanized when grown under laboratory conditions (21). The extensive genetic and phenotypic variability manifested by this fungus led us to hypothesize a comparably high variation in overall secondary metabolite production.

In this study, we explore genomic diversity of three isolates of *Z. tritici* to address the composition of BGC along the fungal genome. We complement genomic analyses with transcriptome and metabolome data to compare potential variations in secondary metabolite profiles of the three field isolates. Interestingly, we find evidence for conserved biochemical profiles of the three isolates, contrasting with the highly variable content and production of effector proteins in this species. Lastly, we analyze secondary metabolite production in a *kmt6* mutant (deletion of the KMT6 histone methyltransferase gene) impaired in catalyzing the histone modification H3K27me3 to assess the relevance of chromatin-based BGC regulation in *Z. tritici*. We provide evidence for extensive chemical differences between the three field isolates of *Z. tritici* and show that histone modifications in this fungus, in contrast to other fungi, such as species of Fusarium and Aspergillus (15, 22–24), play a minor role in regulating secondary metabolite production during axenic growth.

## RESULTS

### *In silico* predictions and comparison of BGCs among three field isolates of *Z. tritici*.

**FIG 1:**
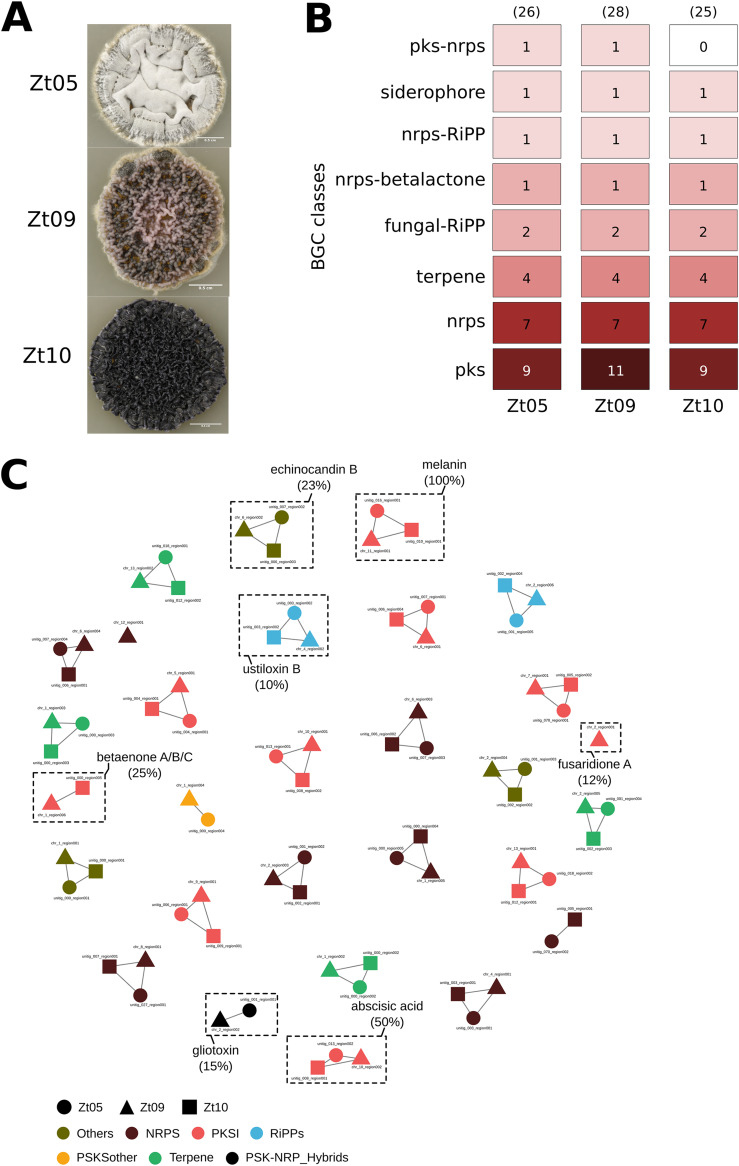
Mining the genome of three *Z. tritici* field isolates for biosynthetic gene clusters. (A) Differences in the colony morphology between the three *Z. tritici* isolates (i.e., Zt05, IPO323, and Zt10) after 15 days of growth on solid medium (YMS) at 18°C. (B) Output from antiSMASH analysis that predicts biosynthetic gene clusters (BGCs) from the genome of the three *Z. tritici* isolates. The fungal isolates Zt05, IPO323 (Zt09), and Zt10 are predicted to harbor 26, 28, and 25 BGCs, respectively. These BGCs are categorized into eight classes, including polyketide synthase (pks), nonribosomal peptide synthetase (NRPS), terpene, ribosomally synthesized and posttranslationally modified peptides (fungal-RiPPs), siderophore, and hybrid NRPS-betalactone, NRPS-RiPP, and PKS-NRPS clusters. (C) Similarity network of BGCs predicted from Zt05 (circle), IPO323 (triangle), and Zt10 (square). Each node in the network (i.e., circle, square, or triangle) depicts a BGC, and the color indicates the class. BGCs connected by edges belong to the same biosynthetic gene family. Seven BGFs (highlighted with a dashed rectangle) showed percentages of gene similarity to annotated clusters in the MiBIG database.

To compare the genomic potential for secondary metabolite production among isolates of *Z. tritici*, we used the program antiSMASH (25) to predict the distribution of BGCs in high-quality genome assemblies of three field isolates, Zt05, IPO323, and Zt10 (18). We have previously described and compared the infection development of Zt05, IPO323, and Zt10 in susceptible wheat and demonstrated that these isolates, although morphologically and genetically highly distinct, are equally virulent in a susceptible wheat cultivar (26). Interestingly, these three isolates exhibit an exceptional phenotypic diversity when cultivated on agar, whereby the isolate Zt10 appears highly melanized, in contrast to the two other isolates (Fig. 1A).

Based on the *in silico* prediction, we find that the three isolates overall encode comparable numbers of BGCs (Fig. 1B). In total, we identified 26, 28, and 25 BGCs in the genomes of Zt05, IPO323, and Zt10, respectively. These gene clusters are categorized into several classes, including polyketide synthases (PKS), nonribosomal peptide synthetases (NRPSs), terpene synthase (TPS), fungal RiPPs (ribosomally synthesized and posttranslationally modified peptides), hybrid NRPS-betalactone, NRPS-RiPPs, PKS-NRPSs, and siderophores (Fig. 1B; see also Tables S1 to S3 in the supplemental material).

We next investigated in detail the variation in BGC distribution and sequence composition among genomes of the three isolates. To this end, we calculated the similarity across all predicted BGCs using BiG-SCAPE to further identify shared and unique BGCs within and among the *Z. tritici* isolates (27). The resulting sequence similarity network consists of 29 biosynthetic gene families (BGFs), shown as a subnetwork (Fig. 1C) and representing seven main BGC classes, PKS, NRPS, RiPPs, PKS-NRP hybrids, TPS, other PKS, and others. Almost all BGFs (27) contain two to three BGCs, and none of these BGFs are composed of two BGCs originating from the same isolate (Table S4). We identified two BGCs in IPO323 that show no similarity to other BGCs (using a clustering cutoff of 0.3), indicating that this isolate exhibits to some extent a unique biosynthetic potential. However, the overall *in silico* BGC predictions suggest that the three isolates share overall similar biochemical potentials.

Of all the predicted BGCs, we observed that five, seven, and five BGCs, in Zt05, IPO323, and Zt10, respectively, show similarity to annotated BGCs in the MiBIG database (with a similarity of >10% in terms of gene content and order) (Fig. 1C and Tables S1 to S3). These annotated gene clusters have similarity to gene clusters producing the compounds ustiloxin (10% similarity), fusaridione (12% similarity), gliotoxin (15% similarity), echinocandin (23% similarity), betaenone (25% similarity), absicic acid (50% similarity), and melanin (100% similarity). Several of these compounds are likely to play a role in pathogenicity of *Z. tritici*: betaenone and ustiloxin have previously been described as phytotoxins in the plant-pathogenic fungi *Phoma betae* and *Ustilaginoidea virens*, respectively (28–30). Fusaridione and gliotoxin, mainly described in the plant-pathogenic fungus Fusarium *heterosporum* and the human pathogen Aspergillus fumigatus, respectively, are known to have antimicrobial properties that may facilitate competitiveness of *Z. tritici* with respect to other plant-associated microbes (31, 32). Echinocandins are fungus-produced metabolites that are responsible for antifungal activity against β-(1,3)-d-glucan synthesis (33). Finally, melanin is known to be a critical component of pathogenicity in many plant-pathogenic fungi (34). Our *in silico* prediction moreover identified one BGC present in all three isolates that shows 50% similarity to an abscisic acid (ABA) gene cluster described in Botrytis cinerea (Tables S1 to S3) (35). Abscisic acid is also known as a plant hormone involved in abiotic and biotic stress regulation, and this hormone can act to suppress defense regulation (e.g., see references 36–38). Further testing will help us to determine whether *Z. tritici* produces ABA in order to interfere with plant host defenses during infection.

### Differential regulation of BGCs in three field isolates of *Z. tritici*.

We next asked how secondary metabolite production is regulated during plant infection in the three *Z. tritici* isolates. To this end, we used previously published transcriptome data generated for the three isolates during wheat infection (26). The infection development of *Z. tritici* is characterized by four infection stages previously described with detailed microscopy analyses (26). In brief, stage A represents the initial penetration of the fungus via the stomata and early colonization of the mesophyll tissue. Stage B represents the biotrophic colonization of the mesophyll tissue, stage C represents the transition from biotrophic to necrotrophic growth, and stage D represents the necrotrophic colonization and asexual reproduction.

**FIG 2:**
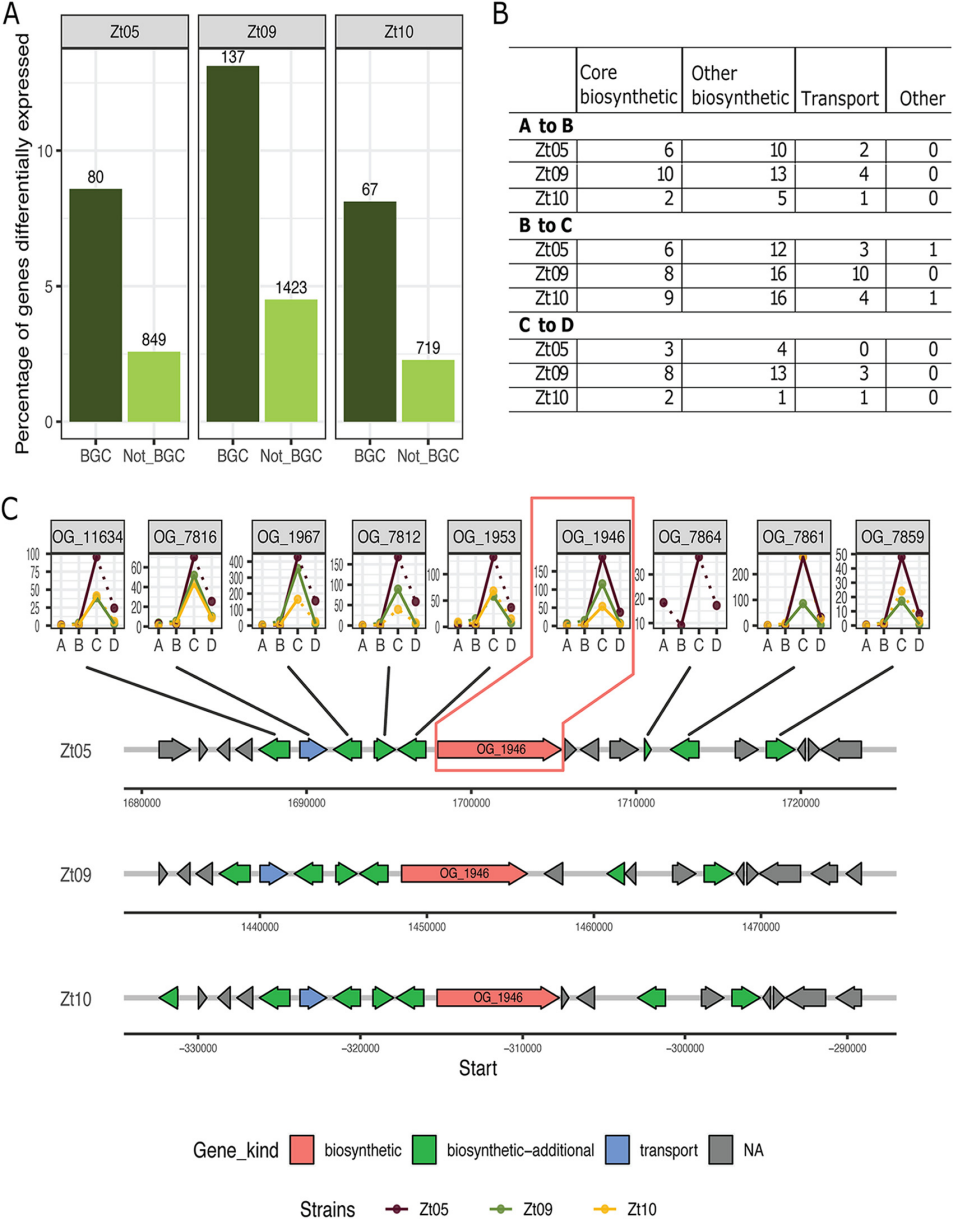
Expression profiles of genes associated with biosynthetic gene clusters in *Z. tritici* isolates. (A) Bar plot representing the percentage of genes differentially expressed during host infection (based on comparison of four infection stages). The numbers above the bars represent the number of differentially expressed genes predicted to be in gene clusters (BGC, in dark green) and all other genes (not BGC, in light green). (B) Numbers of differentially expressed genes in BGCs for each *Z. tritici* isolate during four infection stages. (C) Schematic overview of the genetic architecture of the BGC predicted to produce abscisic acid (ABA). The expression kinetics of the ABA genes in the three isolates are represented as line plots linked to the orthologs in Zt05. On the line graphs, the *y* axis represents the TPM values, while the *x* axis denotes the infection stages. A dotted line represents change in expression level that is not significant, whereas significant differences are represented by a full line. The line plots are labeled with the orthogroup number as identified in reference 18.

We focused our gene expression analyses on the BGCs identified by our *in silico* prediction. To this end, we first showed that the vast majority of predicted BGCs are expressed during plant colonization (between 87% of genes in stage A for Zt10 and 98% in stage D for Zt09 [a clone derived from IPO323]; Fig. S1). Consistent with the small amount of fungal biomass and therefore overall lower proportion of fungal transcripts, we found an increased transcript abundance of all genes, including BGC genes, in the later stages of infection (C and D) (see percentage of alignments increasing in Table S5). Next, we compared the expression specifically of BGC genes in the three isolates throughout infection development to uncover patterns of secondary metabolite production (we compared the differential expression between the consecutive stages A and B but not between stages A and D). This comparison revealed a dynamic expression of BGC genes, with 9%, 13%, and 8% of the BGC genes in Zt05, Zt09, and Zt10, respectively, being differentially expressed between the infection stages (Fig. 2A and Table S6). In comparison, the overall proportions of differentially expressed genes for non-BGCs were consistently lower, 2.6%, 4.5%, and 2.3% for Zt05, Zt09, and Zt10, respectively, suggesting that different BGCs are important during different infection stages of *Z. tritici*.

The genes encoded by individual BGCs have different functions in metabolite synthesis, and we next focused on the expression of individual genes in the BGCs predicted to have a primary function in metabolite synthesis. The program antiSMASH distinguishes core biosynthetic genes, biosynthetic genes, regulatory genes, transporters, and genes with unknown functions for individual BGCs. We focused specifically on the core biosynthetic genes from the predicted BGC using previously defined orthogroups (18) to compare expression patterns between isolates (Fig. 2B). In total, we identified 44 core biosynthetic gene orthogroups among the 29 BGCs (found in at least one strain). Of note, a few clusters contain more than one core biosynthetic gene, and some clusters were identified in only one or two of the isolates. We investigated the expression kinetics of these genes throughout the four infection stages and revealed a highly dynamic expression pattern of the core BGC genes (Fig. S2, Table S7). In particular, we observed a general pattern of higher gene transcription during stages C and D, i.e., the necrotrophic phase, an increase in transcription that is significant in several core biosynthetic genes (see, for example, BGFs 8, 9, 15, 18, 20, and 24 in Fig. S2).

We also identified differences in expression of BGC genes among the three isolates. For example, one gene in the BGC encoding the phytotoxin betaenone (BGF5:OG_10647) showed increased expression in the Zt10 isolates during the transition from biotrophic to necrotrophic host colonization, suggesting differential relevance of the metabolite among the three *Z. tritici* isolates. We compared the expression profiles of the seven genes predicted by antiSMASH that compose the gene cluster BGF_11 (ustiloxin; 10% similarity). Overall, the genes were expressed in the same manner in the three isolates. Six of the seven ustiloxin biosynthesis genes showed an increased expression during stage B and C, suggesting that this phytotoxin plays a role in the transition from biotrophic to necrotrophic host colonization. On the other hand, one gene, BGF 11:OG_11282, shows a low expression during stages A to C but then a strong upregulation during the late phase of infection and necrotrophic host colonization. The biosynthetic genes clusters BGF_28 (fusaridione 12% similarity) and BGF_29 (gliotoxin 15% similarity) did not show consistent expression patterns during host colonization across the three field isolates (i.e., Zt10, Zt09, and Zt05). The gene BGF_28:OG_5759, putatively involved in fusaridione biosynthesis, showed a decreased expression from stage A through D in Zt09. The gene BGF_29:OG_2829 showed an increased expression in Zt05 but a more dynamic and inconsistent pattern in Zt09. Three genes (out of four) of BGF17 (echinocandin; 23% similarity) showed upregulation during early biotrophic host colonization across the three isolates, while one gene (i.e., BGF_17:OG_5907) was downregulated in the fungal isolate Zt09. One gene predicted to be involved in melanin biosynthesis and carried by BGC_23 showed an increased expression in all three isolates throughout infection development (stages A to D), possibly reflecting protection of the fungal cell wall during infection establishment and progression.

One gene showing a strong upregulation in the three isolates during the transition from biotrophic to necrotrophic growth (i.e., phase C) belongs to BGF_20, predicted to be responsible for the production of ABA (BGC showing 50% gene similarity to the abscisic acid gene cluster in Botrytis cinerea). Given the potential role of ABA in pathogenicity, we further investigated the genetic architecture of the ABA BGC in *Z. tritici* and transcription among the three isolates as well as the predicted function of each gene (Fig. 2C and Table S8). Overall, we found that the synteny and gene functions of the predicted ABA BGC are conserved among the three isolates. Eight genes with a predicted role in cluster function were shared in the three isolates and have the same order; however, one additional gene was found in the BGC of Zt05 and another in Zt10 (Fig. 2C). The expression kinetics of the genes in the ABA cluster predicted to have a function in compound synthesis and in transport showed a common upregulation at the onset of the necrotrophic phase (significant upregulation in two isolates for all shared orthologs and significant in all three isolates for the core biosynthetic gene) (Fig. 2C and Tables S5 and S6).

The expression patterns of the ABA genes, which we observed *in planta*, suggest that ABA is produced by *Z. tritici* during host infection to manipulate plant defenses. We further hypothesize that the other BGC genes, which show a similar kinetics, likewise play an important role during host colonization.

### Variation in secondary metabolite profiles of *Z. tritici* field isolates.

We next sought to characterize the chemical profile of the three field isolates during axenic growth. To this end, we applied a comparative untargeted metabolomics approach using tandem mass spectrometry (MS^2^)-based molecular networking (MN). MN is a comprehensive algorithm-based metabolomics tool that compares MS^2^ spectra and categorizes them into different structural clusters based on fragmentation patterns (39). A recently developed tool, feature-based molecular networks (FBMN), incorporates MS-related information, such as retention time and isotope patterns, into data processing, thereby facilitating the annotation of molecular families (MF) and allowing for resolution of positional isomers and relative quantification (39–41). Here, we applied FBMN analyses that assisted us in determining and comparing the secondary metabolite profile of the three *Z. tritici* field isolates.

**FIG 3:**
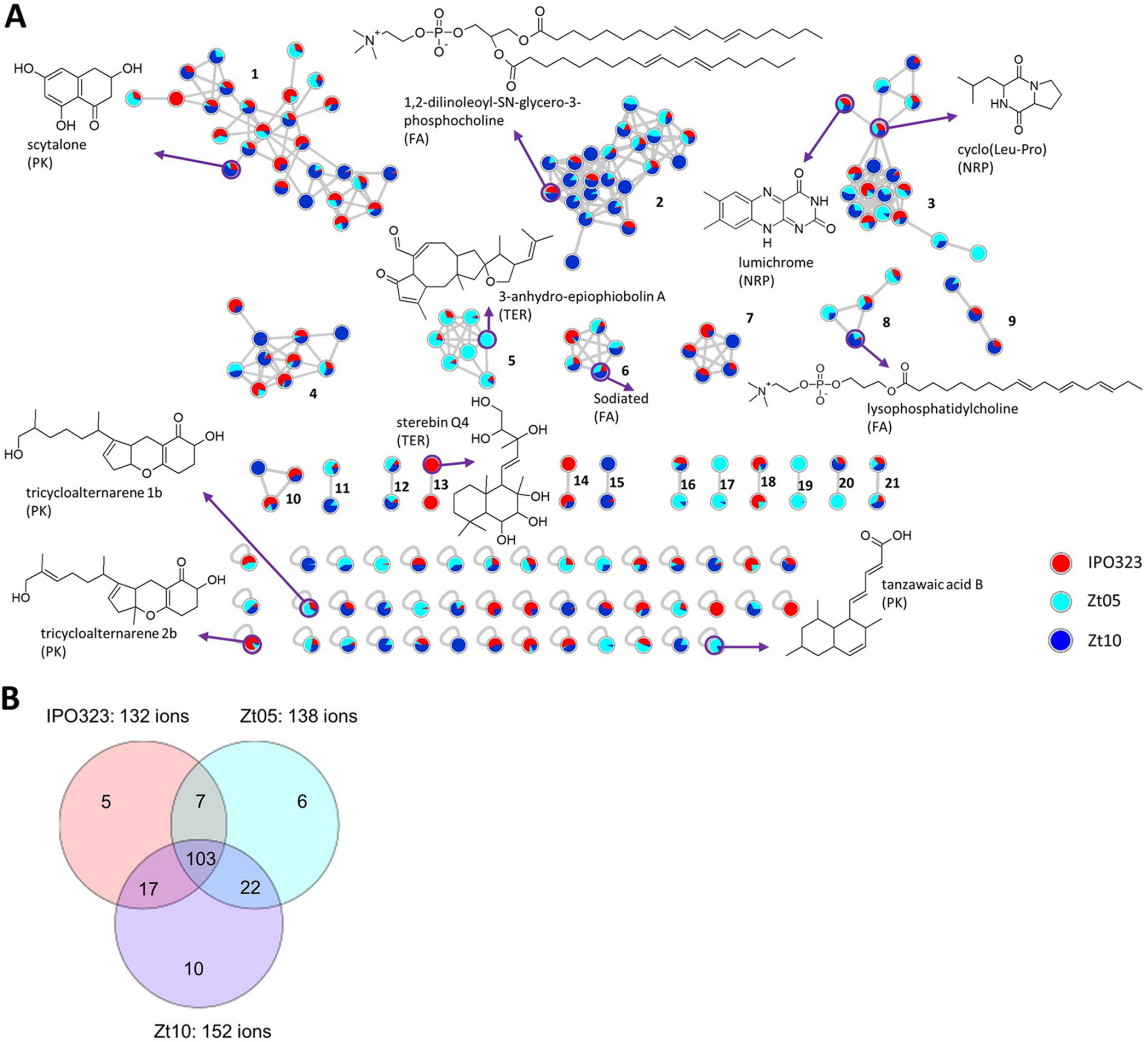
Comparative metabolomics of three *Z. tritici* field isolates. (A) Molecular network (MN) generated from the Global Natural Product Social Molecular Networking platform of crude organic extracts from the three isolates of *Z. tritici*. Nodes represent molecular ions detected in the crude extracts, with color coding indicative of the relative abundance in each *Z. tritici* species (red, IPO323; light blue, Zt05; deep blue, Zt10). Some annotated compounds are displayed with their chemical classes: polyketide (PK), nonribosomal peptide (NRP), terpene (TER), and fatty acid (FA). Other annotations are displayed in Table S8. (B) Venn diagram displaying specific and shared parent ions detected in the culture extracts of the three *Z. tritici* species.

The metabolome analyses of the *Z. tritici* field isolates revealed a total of 170 of the so-called nodes, each representing one molecular ion in the crude metabolite extracts (see Materials and Methods). We grouped these into 21 clusters and 43 singletons (Fig. 3A). Overall, five putative metabolite clusters were annotated as polyketide (PK), fatty acid (FA), nonribosomal peptide (NRP), and terpene (TER) MFs, as also identified in the genome analyses. The data from MN were used to generate a Venn diagram to visualize the metabolite distribution among the isolates (Fig. 3B). We find that Zt05 displayed 138 ions, IPO323 132 ions, and Zt10 152 ions. Twenty-one ions were exclusively found in one isolate, namely, 10 ions for Zt10, six ions for Zt05, and five ions for IPO323, respectively (Fig. 3B and Table S9). These strain-specific ions and the different expression levels of common ions could be responsible for individual strain variation in biochemical profiles, for example, the very prominent variation in melanization (Fig. 1A). Conversely, a total of 103 ions were common to all field isolates, while 46 ions were shared by two strains (Fig. 3B). These data indicate that the field isolates overall produce similar metabolites when maintained under the same culture conditions.

As shown in Fig. 3A, a combined automated and manual dereplication effort resulted in the putative annotation of the polyketide scytalone (42), the diketopiperazine (NRP) cyclo-Leu-Pro (43), the labdane diterpene sterebin Q4, and the phospholipids lysophosphatidylcholine and 1,2-dilinoleoyl-SN-glycero-3-phosphocholine. Other annotated compounds included three PKs, the benzopyranones trycycloalternarene 1b and tricycloalternarene 2b (44), the bicyclic tanzawaic acid (45), and the sesterterpene 3-anhydro-6-epiophiobolin A (46).

Scytalone, a naphthalenone type PK, was present in all three strains and formed the largest cluster, cluster 1, in the FBMN (Fig. 3A). This compound is a known intermediate in melanin biosynthesis, and the presence of this compound concurs with the presence of the BGC responsible for this metabolite in the genome data (Fig. 1C). Its differential expression levels in the three strains, highest in Zt10 and lowest in ZT05 (determined by peak areas in Table S9), also explain the variation in melanization (Fig. 1A). The cluster of nodes around scytalone (cluster 1) included ions predicted as polyketides. However, manual and automated dereplication efforts did not result in further annotation of melanin-related metabolites. The polyketide cluster 1 included 27 ions shared by all three strains. In this cluster, only two ions seen as blue only (*m/z* 323.3149 [M+H]^+^) and red only (*m/z* 355.2904 [M+H]^+^) were specific to Zt10 and IPO323, respectively. We were not able to annotate these isolate-specific ions of polyketide origin, despite the use of multiple databases.

In the second largest cluster (cluster 2), 1,2-dilinoleoyl-SN-glycero-3-phosphocholine (primary metabolite) was annotated as belonging to a class of fatty acid (FA) compounds (Fig. 3A) (47). This MF had 23 ions with many putative positional isomers, which, however, could not be annotated. Some sodiated ions were annotated as FAs in cluster 6 (Fig. 3A) and were also observed in the FBMN.

Another class of compounds annotated in the *Z. tritici* metabolite network was the nonribosomal peptides of cluster 3. We annotated a class of diketopiperazines, including cyclo-Leu-Pro, cyclo-Val-Leu (Fig. 3A and Fig. S5 and Table S9), and the alloxazine lumichrome (Fig. 3A). Cluster 5 was shared by all three *Z. tritici* isolates, with 2 ions specific to Zt10. One of these ions could be annotated to be a sesterterpene, 3-anhydro-6-epiophiobolin A (Fig. 3A).

Overall, ∼30% of the detected ions could be annotated as known fungal metabolites, while the majority remain putative new compounds have yet to be described (Table S9). There was a negligible difference in the expression of compounds among the three field isolates (20/21 MFs shared across the three *Z. tritici* isolates). Although Zt10 produced more ions (152 ions), IPO323 displayed more diverse chemistry and produced the only isolate-specific MF (cluster 13). This cluster comprises an ion, annotated to be a labdane diterpenoid sterebin Q4 (Fig. 3A), and two unannotated IPO323-specific singletons, *m/z* 373.2582 [M+H]^+^ and *m/z* 388.2692 [M+H]^+^, which may define a specific biochemical profile of the isolate. Taken together, our metabolomic study shows that the three isolates show comparable metabolite profiles *in vitro* and may harbor untapped molecules with biological functions.

### Removal of the histone modification H3K27me3 has little effect on BCG expression and metabolite production.

Previous studies have revealed a prominent role of histone modifications in the regulation of fungal virulence factors, including effectors and secondary metabolites (14, 40). We used a *Z. tritici* mutant deficient in the trimethylation of histone H3 lysine 27 (H3K27me3) (48) to address if this histone mark likewise plays a prominent role in regulation of the identified BGCs in the *Z. tritici* genome. To this end, we reanalyzed available transcriptome data (48) to assess the extent of differential gene expression between wild-type *Z. tritici* and the IPO323Δ*kmt6* methyltransferase mutant, and we compared the metabolome profiles of the wild type and mutant.

As previously reported (48), wild-type *Z. tritici* and the *kmt6* mutant do not differ substantially in gene expression (381 genes were found to be differentially expressed, whereas 10,169 genes did not show any significant difference in expression between the wild type and the mutant) (Table S10). Among the differentially expressed genes, only six are associated with the BGCs that we describe in this study, including a predicted transporter and an additional biosynthetic gene. None of these differentially expressed genes were predicted to be core biosynthetic genes. These results support previous findings suggesting that H3K27me3 does not play a prominent role in the regulation of secondary metabolite biosynthesis in *Z. tritici* (48).

**FIG 4:**
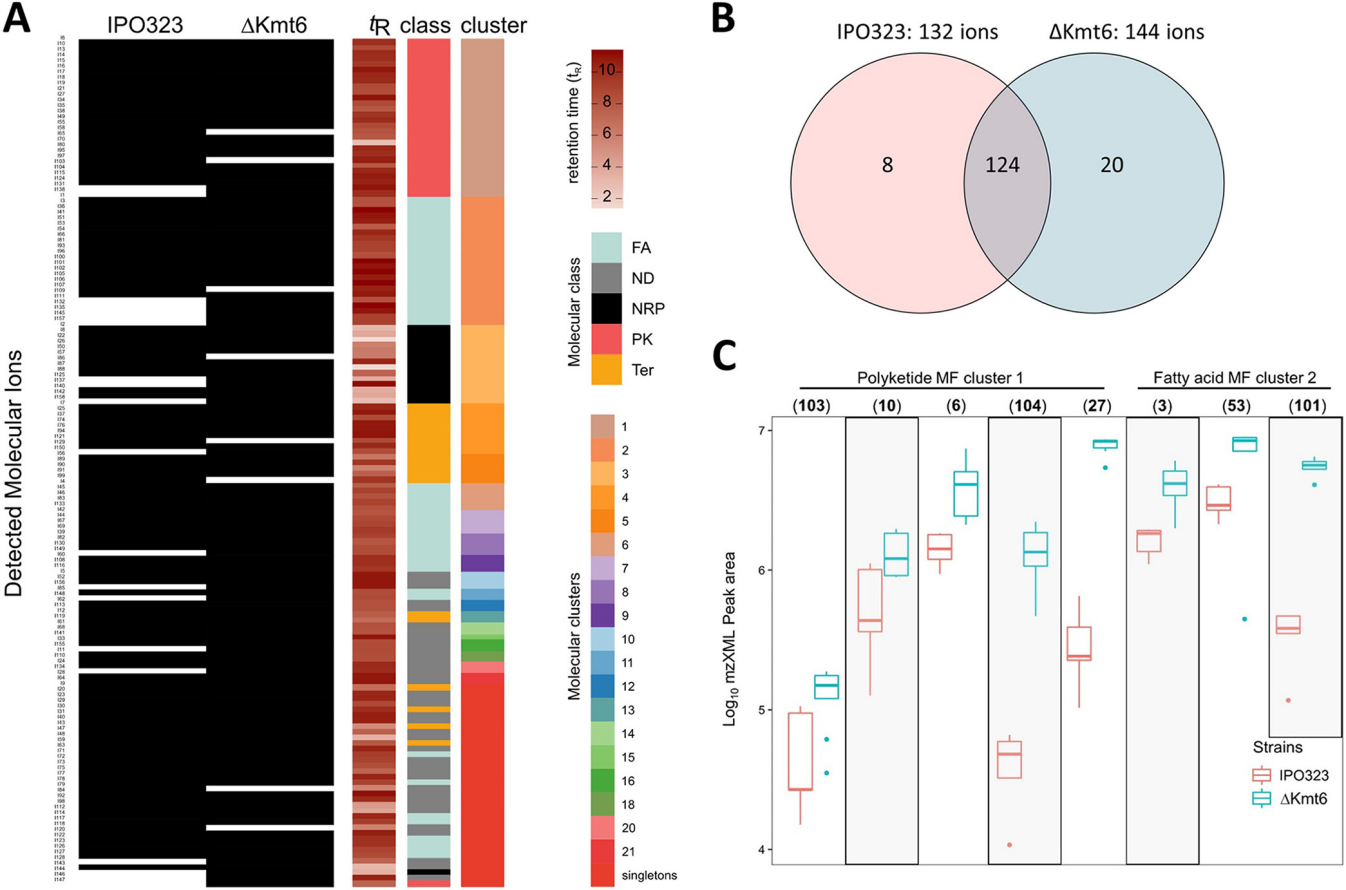
Comparative metabolomics of the wild strain IPO323 and Δ*kmt6* mutant. (A) Presence-absence heat map from the UPLC-MS analyses of IPO323 and Δ*kmt6* organic extracts showing the distribution of *m/z*, retention time, chemical classes (FA, fatty acid; NRP, nonribosomal peptide; PK, polyketide; TER, terpene; and ND, not determined), and molecular family clusters. (B) Venn diagram displaying specific and shared parent ions detected in the culture extracts of the IPO323 and Δ*kmt6* mutant. (C) Box plots depicting the relative abundance of distinct molecular ions, in PK MF cluster 1 and FA MF cluster 2, between IPO323 and Δ*kmt6* mutant of *Z. tritici*. Numbers in parentheses represent unique ion IDs, as shown in Table S8: (103), *m/z* 395.2790 [M+H]^+^; (10), *m/z* 321.2422 [M+H]^+^; (6), *m/z* 379.3354 [M+H]^+^; (104), *m/z* 397.2959 [M+H]^+^; (27), *m/z* 323.2582 [M+H]^+^; (53), *m/z* 518.3245 [M+H]^+^; (3), *m/z* 520.3400 [M+H]^+^; (101), *m/z* 784.5837 [M+H]^+^.

To further investigate the role of facultative heterochromatin in the regulation of secondary metabolite production in *Z. tritici*, we compared the metabolomic profile of the wild-type isolate IPO323 to the *Δkmt6* mutant, impaired in trimethylation of the H3 lysine 27. Based on the FBMN, we generated the same set of MFs as those produced for the field isolates (Fig. S3). The data were visualized in a presence-absence heatmap of all the detected molecular ions and their corresponding retention times and molecular clusters (Fig. 4A). Altogether, 152 ions were produced and organized into 18 MFs (clusters) with 39 singletons (Fig. 4A and Fig. S3). Importantly, no strain-specific MF was produced by the two strains, which confirms the transcriptome data and also indicates that H3K27me3 plays a minor role in regulating specific secondary metabolites in this fungus. Notwithstanding this finding, the *Δkmt6* mutant produced 144 ions with 20 specific ions, and the wild type produced 132 ions with 8 specific ions (Fig. 4B). The majority of the ions, 124, representing approximately 81% of ions produced, were common to both strains (Fig. 4B).

Application of FBMN leveraged the MZmine feature detection and alignment tool to allow for relative quantification of peak ions produced by IPO323 and the *Δkmt6* mutant. Table S9 summarizes the detected ions, their unique identifiers (IDs), and their relative abundance (peak areas) to assist comprehensive comparison of production titers of the major metabolites by the *Z. tritici* wild-type and *Δkmt6* strains. Peak area box plots were generated for distinct ions detected in clusters 1 (polyketides) and 2 (fatty acids) to ascertain the effect of H3K27me3 on increasing the production titers of some compounds in the mutant strain. Several molecular ions from the polyketide MF cluster 1 showed an increased abundance in the Δ*kmt6* mutant strain (Fig. 4C and Table S9). This included 103 (*m/z* 395.2790 [M+H]^+^), 10 (*m/z* 321.2422 [M+H]^+^), 6b (*m/z* 379.3354 [M+H]^+^), 104 (*m/z* 397.2959 [M+H]^+^), and 27 (*m/z* 323.2582 [M+H]^+^. In fatty acid cluster 2, molecular ions 3 (*m/z* 520.3400 [M+H]^+^), 53 (*m/z* 518.3245 [M+H]^+^), and 101 (*m/z* 784.5837 [M+H]^+^) were also expressed in relatively higher quantities in the *Δkmt6* mutant than in the IPO323 wild type (Fig. 4C). Overall, about 95% of the detected molecular ions were shared by the two strains with very minimal differences in terms of genes expressed under the specified cultivation conditions. In summary, while H3K27me3 does not contribute to the expression of specific gene clusters and the production of individual metabolites, it enhances the production of several compounds quantitatively in the polyketide and fatty acid-derived MFs.

## DISCUSSION

The fungal species *Z. tritici* is one of the most devastating pathogens of temperate-grown wheat and causes severe yield losses to farmers in addition to high costs of crop protection measures. A challenge to the development of sustainable control strategies is the high level of genetic variation in field populations of *Z. tritici* that enables the fungus to rapidly adapt to changes in its environment. Genome and transcriptome studies have demonstrated that infection of *Z. tritici* relies not on major virulence determinants but on a large repertoire of effector proteins that are produced to facilitate host invasion. Interspecific hybridization has occurred repeatedly in *Z. tritici* and has been demonstrated to be an important mechanism of gene exchange between species as well as a source of within-species variation (49, 50). In addition to proteinaceous virulence determinants, fungal pathogens also produce secondary metabolites during host infection. We addressed here whether the secondary metabolite profiles of three field isolates of *Z. tritici* also show a high variability consistent with the high variation of effector gene content and expression of the same isolates (26). We designed a pipeline for *in silico* prediction of genes in three high-quality genome assemblies of *Z. tritici* isolates (18). Based on these complete or nearly complete chromosome assemblies, we found very similar numbers of BGCs among the three isolates. Our updated prediction in this study provided a slightly lower number of BGCs than previously reported for the reference isolate IPO323 (29 versus 32), but it was comparable to predicted numbers of BGCs in other Dothideomycete genomes (51). The conserved composition of BGCs in three field isolates of *Z. tritici* suggest that this pathogen does not rely on diverse chemistry as, e.g., pathogenic and endophytic lineages of the plant-associated fungus *Epichloë* (9) but rather on a conserved repertoire of metabolites.

Further annotation identified putative functions of some *Z. tritici* BGCs. Two of the predicted clusters show similarity to BGCs identified in other fungi and known to be involved in the production of the phytotoxins ustiloxin and betaenone. We find that genes located in BGF_11 (ustiloxin; 10% similarity) are upregulated during host infection. It is plausible that the product of this BGC plays a role during *Z. tritici* host colonization. The effect of ustiloxin has been documented on cell cultures and rice seedlings, and the compound shows a variety of biological activities, including antimitotic activity, whereby it can inhibit microtubule assembly (52). The relevance of this compound in the *Z. tritici*-wheat interaction is not known.

Two *Z. tritci* isolates were predicted to carry a BGC that shows 25% similarity to the betaenone gene cluster (i.e., BGF5). We have indicated that only one gene in this cluster is upregulated, and it was exclusively observed in the fungal isolate IPO323. Betaenones A to C are known polyketides with phytotoxicity and are produced by the sugar beet pathogen *P. betae* (53). Noar and colleagues have reported that the banana pathogen *Pseudocercospora fijiensis* carried a gene cluster, PKS8-4, that had similarity to the betaenone gene cluster of *P. betae*, further suggesting that this BGC produces a metabolite with a structure similar to that of betaenone (54). Interestingly, the expression of this BGC was specific to spermagonia, and its disruption did not alter the pathogenicity of *P. betae*. Based on our expression profiles and the findings of Noar and colleagues, we hypothesize that the product of BGF5 in *Z. tritici* has less relevance during host infection. Further, our analysis revealed that both fungal isolates harbor BGCs that encode proteins involved in the synthesis of melanin. Melanin is encoded by broadly diverse fungal species and has been shown to have a critical role in fungal survival under stress conditions (see, e.g., a review in reference 55) and play, broadly, a role in fungal pathogenicity (34). However, melanin-deficient strains of *Z. tritici* were not altered in their virulence or host colonization under test conditions (56), suggesting that melanin in *Z. tritici* plays other critical roles that require further testing.

We also show the upregulation of genes in BGCs predicted to synthesize the antimicrobial and antifungal compounds gliotoxin, fusaridione, and echinocandin. Recent studies have drawn attention to the interaction of plant pathogens with their host microbiota (57, 58). Fungal plant pathogens may produce a multitude of compounds to compete and exclude coexisting microorganisms in their host tissues. A recent study in the wilt pathogen *Verticilium dahliae* found an effector protein secreted by the fungus to manipulate host microbiota composition (58). We predict that *Z. tritici* likewise produces antimicrobial and antifungal compounds to reduce growth of competing bacteria and fungi.

The gene cluster BGF_23 is predicted to be responsible for the synthesis of abscisic acid (ABA). Genes in this cluster show differential regulation across infection stages and are significantly upregulated in all three isolates during the transition from biotrophic to necrotrophic growth. ABA is primarily known as a plant hormone playing a role in plant development and response to abiotic stresses. Environmental stresses, including drought, increased salinity, and increased temperature, are cues that induce the production of ABA. However, ABA also has an antagonistic effect on jasmonic acid (JA) signaling and thereby reduces defense signaling (38). Exogenous ABA was found to confer a strong increase in susceptibility of tomato seedlings toward the pathogen Botrytis cinerea (59). In the rice pathogen Magnaporthe oryzae, ABA was found to be a necessary component of fungus virulence (60), and ABA has been qualified as an effector in both plant and animal pathogens (61). It is worth mentioning that *Leptosphaeria masculans* produces ABA, and the deletion of two genes involved in the production of ABA in this fungus did not alter its pathogenicity on canola. Further functional characterization of the gene BGF_20:OG_1946 (ABA; 50% similarity) would shed light on whether this metabolite plays a role during host colonization by interfering with induced defense signaling in the pathogen *Z. tritici*. Genetic tools are available for *Z. tritici*, allowing the deletion of larger fragments of DNA or individual genes by *Agrobacterium*-mediated transformation (21). Deletion of the ABA gene cluster or the core ABA gene will allow a detailed assessment of the relevance of ABA production in successful host colonization of *Z. tritici*.

We applied metabolomics to characterize the biochemical profile of the three field isolates when propagated axenically. Consistent with the *in silico* prediction, we observed highly similar metabolome profiles in the three field isolates (Fig. 3B). Molecular networking revealed a large number of MFs common to all isolates. Although the overall metabolome profiles agree with the presence of BGCs predicted to produce these metabolites, there were other pathways for which the expected compounds were not detected. It is well known that a majority of fungal BGCs are silent during cultivation in an artificial medium in the laboratory (62). Production and detection of the secondary metabolites rely on (i) its expression that may be influenced by the culture conditions, (ii) extraction techniques, and (iii) production levels of the compounds, which must be above the limit of detection for mass spectrometry or threshold employed for automated feature detection. The lack of detection and annotation of ABA in the *in vitro* extracts, as deduced by both positive- and negative-mode high-resolution MS (HRMS) and HRMS/MS analyses, plus ^1^H nuclear magnetic resonance (NMR) spectroscopy (Fig. S7), is consistent with the low expression of genes in the ABA cluster outside the plant (63). We speculate that certain host cues induce the expression of the ABA BGCs during host infection. Interestingly, our metabolomics study also revealed some putative metabolites whose BGCs were not characterized in the genome analysis. Known phytotoxins such as trycycloalternarenes and ophiobolins, previously isolated from Dothideomycetes (64, 65), were not predicted in the *Z. tritici* genome data, although BGCs encoding these compounds were found in the metabolite extracts. An explanation for this discrepancy is the lack of information in the databases to confidently predict the genes encoding these metabolites in genome data.

In some fungal species, heterochromatin plays an important role in the regulation of secondary metabolites. In Aspergillus nidulans, loss of function of *cclA*, a component of the COMPASS complex, which methylates lysine 4 on histone H3, activated two pathways to produce the anthraquinone cluster of compounds monodictyphenone and emodin and the polyketides F9775A and F9775B (66). Here, we included the IPO323Δ*kmt6* mutant, which is impaired in the trimethylation of H3 lysine 27. While H3K27me3 is a global regulator of secondary metabolite production in F. graminearum, it appears to play a minor role in the regulation of BGCs in *Z. tritici*. This concurs with our previous transcriptome sequencing (RNA-seq) analyses of the same mutant showing a minor difference in transcriptional regulation (48). Rather, this histone modification plays a central role in the stability of accessory chromosomes in *Z. tritici* (48). It has been observed that in addition to awakening BGCs, which are otherwise silent (66, 67), histone modification can impact the quantitative production of constitutive secondary metabolites. For example, the loss of *cclA* gene in Aspergillus oryzae enhanced the overproduction of the sesquiterpenes astellolides (68). The plant endophytic fungus *Pestalotiopsis fici cclA* mutant also increased the production of pestaloficiols and macrodiolide polyketides (69), providing further evidence for the relevance of histone modifications in secondary metabolite regulation in many fungal species. In *Z. tritici*, secondary metabolites are produced in the histone mutant; however, we observed quantitative differences in the relative abundance of some metabolites whereby some metabolites are produced in higher quantities in the IPO323Δ*kmt6* mutant (Fig. 4C). This finding suggests that some regulation occurs either directly or indirectly via H3K27me3, although not a main regulatory mechanism.

In conclusion, we find that the genome of the wheat fungal pathogen *Z. tritici* carries BGCs that may produce an untapped diversity of metabolites. Our study demonstrates that the three *Z. tritici* isolates have similar repertoires of BGCs that contrast the high variability in gene expression patterns, notably of effector genes and morphological phenotypes. Further, the in-depth analysis of the transcriptional profiles of the annotated BGCs, as well as metabolomic profiles, did not reveal striking differences between the three field isolates, indicating that the three *Z. tritici* isolates share an infection program. In contrast to F. graminearum, the histone methylation mediated by the histone methyltransferase Kmt6 plays a minor role in regulating the expression of BGCs and production of secondary metabolites. This study revealed that *Z. tritici* has BGCs that potentially interfere with plant immunity and compete against the host-associated microbiome. Understanding at which infection stage these metabolites are produced will help to further decipher their function *in planta* and better understand the infection process of this pathogen.

## MATERIALS AND METHODS

### Genome data and BGC identification.

To predict biosynthetic gene clusters (BGCs), the fungal genomes were submitted to antiSMASH fungi 4.0.2 (25). The Jaccard similarity network of BGC families was constructed using BiG-SCAPE (v1.0.0; cutoff distance, 0.3) by mixing all classes (all versus all) (27). The network was plotted using R function ggnet2 from the GGally package (https://ggobi.github.io/ggally). AntiSMASH analysis output can be accessed at https://zenodo.org/record/4592481. Gene function predictions were obtained from the data published in reference 18.

### RNA-seq analyses.

All transcriptomic data sets used in this study were previously published (26, 48). We reanalyzed the raw data with a focus on current annotation of BGCs. Adapter removal and read trimming were done with Trimmomatic v0.38 with parameters LEADING: 30 TRAILING:30 MINLEN:30 (70). The alignment of the trimmed reads to each genome assembly (IPO323, Zt05, and Zt10) was done with HISAT2 version 2.2.1 with intron length set between 20 and 15,000 (71) and read counting with HTseq v0.11.2, both steps set with the option for reverse-strand orientation (72). Genes annotated previously (18) as being reverse transcriptase or transposons were filtered out. Genes were defined as not detected when no reads aligned to them. Differential expression analyses were realized with the DESeq2 R package (73). Thresholds for defining significance of differential expression were an adjusted *P* value of ≤0.001 and log fold change of >2. The normalized expression was computed as transcripts per million (TPM) from the read counts based on the exonic gene length and averaged over replicates for plots used in figures here. All scripts for the alignments, the TPM, the differential expression, and the plots generated for the RNA-seq analyses can be found in the supplemental material.

### Fungal extraction.

In this study, we used three *Z. tritici* isolates that were previously described (26). Previous studies using axenic propagation of *Z. tritici* have used fungal cells propagated on yeast-malt-sucrose (YMS) medium (21), and similar growth conditions were used in this study. YMS-agar medium was prepared with 0.4% (wt/vol) yeast extract (Bacto yeast extract; Thermofisher), 0.4% (wt/vol) malt extract, and 0.4% (wt/vol) sucrose supplemented with 2% (wt/vol) Bacto agar. The fungal isolates were incubated at 18°C for 15 consecutive days prior to extractions.

We pooled four solid cultures of *Z. tritici* and extracted metabolites with 400 mL ethyl acetate (EtOAc) (Pestinorm; VWR Chemicals, Leuven, Belgium) after homogenizing by an Ultra Turrax at 19,000 rpm. Each EtOAc extract was washed twice with 200 mL of Milli-Q (Arium, Sartorius) water in a separatory funnel to remove salts. The EtOAc layer was then evaporated to dryness by a rotary evaporator (150 rpm at 40°C). Dried extracts were solubilized in methanol and filtered (0.2-μm filter) into storage vials and dried *in vacuo*. Each fungal strain was extracted in triplicate (biological replicates).

### UPLC-QToF-MS analysis.

Chromatograms were acquired on an Acquity ultrahigh-performance liquid chromatography I-class system coupled to a Xevo G2-XS quantitative time of flight (QToF) mass spectrometer (Waters, Milford, MA, USA). Fungal extracts at concentrations of 1 mg/mL were chromatographed on an Acquity UPLC high-strength silica T3 column (C_18_, 1.8 μm, 100 by 2.1 mm; Waters) at 40°C at a flow rate of 0.6 mL/min and injection volume of 0.5 μL in triplicate. A dual-solvent system was comprised of a mobile phase A, 99.9% Milli-Q water–0.1% formic acid (UPLC/MS grade), and phase B, 99.9% acetonitrile (MeCN; UPLC/MS grade; Biosolve BV, Dieuze, France)–0.1% formic acid, at a flow rate of 0.6 mL/min. The gradient was held at 1% B over 0.5 min, increased to 99% B over 11 min, held at 99% for 3 min, back to the starting condition over 0.5 min, and maintained for 2.5 min.

MS and MS^2^ spectra were acquired in data-dependent analysis (DDA) mode with an electrospray ionization source in positive and negative modes using the following parameters: a mass range of *m/z* 50 to 1,200 Da, capillary voltage of 0.8 kV (1 kV in negative polarity), cone and desolvation gas flow of 50 and 1,200 liters/h, respectively, source temperature of 150°C, and desolvation temperature at 550°C with sampling cone and source offset at 40 and 80, respectively. Collision energy (CE) was ramped: low CE from 6 to 60 eV to high CE of 9 of 80 eV. As controls, solvent (MeOH) and EtOAc extract of sterile culture medium were injected under the same conditions. Only the data obtained from the positive mode were further analyzed, because the chromatograms revealed more complex profiles than in the negative mode.

### Molecular networking analysis of *Z. tritici* metabolites.

UPLC-MS/MS data were converted to mzXML format using Proteo Wizard msconvert (version 3.0.10051; Vanderbilt University, Nashville, TN, USA) (74) and then processed with MZMINE 2.33 (75, 76). The mass detection was set to a noise level of 1,000 for MS and 50 for MS^2^. The chromatogram was built with ions showing a minimum time span of 0.1, minimum height of 3,000, and *m/z* tolerance 0.01 (or 10 ppm). The chromatogram was deconvoluted with the baseline algorithm (minimum peak height, 3,000; peak duration, 0.01 to 3; and baseline level, 1,000). Deisotoping of the chromatogram was achieved by the isotope peak grouper algorithm with *m/z* tolerance of 0 (or 10 ppm) and retention time tolerance of 0.2 min. All samples were combined in a peak list using the join aligner algorithm; the data were duplicate peak filtered and ions detected in the solvent, and culture medium blanks were removed from the mass list. Only data with MS^2^ scans and within a retention time range (0 to 11.5 min) were exported as csv (feature quantitative table) and mgf files and uploaded to the GNPS (39) platform for feature-based molecular networking analysis (41). The preprocessed data were filtered by removing all MS^2^ fragment ions within ±17 Da of the precursor *m/z* value. MS^2^ spectra were filtered by choosing only the top 6 fragment ions in the ±50-Da window throughout the spectrum. The mass tolerances were set to 0.05 Da for both precursor ion and fragment ion. A molecular network was then created where edges were filtered to have a cosine score above 0.7 and more than 6 matched peaks. Finally, the maximum size of a molecular family was set to 100, and the lowest-scoring edges were removed from molecular families until the molecular family size was below this threshold. The spectra in the network were then searched against GNPS spectral libraries (39, 77). The library spectra were filtered in the same manner as the input data. All matches kept between network spectra and library spectra were required to have a score above 0.6 and at least 6 matched peaks. Cytoscape data were exported to Cytoscape version 3.6.14 (78) to visualize the networks with nodes representing peak ions and edges representing similarities between peak ions. Molecular formula predictions were done with MassLynx version 4.1. for annotation of parent ions. Predicted molecular formulae were searched against databases such as Dictionary of Natural Product (http://dnp.chemnetbase.com), NP Atlas (https://www.npatlas.org), and SciFinder (https://scifinder.cas.org). Dereplicated peak ions were further checked by comparing the experimental fragments to *in silico* fragments generated from the CFM-ID web server (79). All annotations were assigned confidence levels from 1 to 4 based on the four levels of metabolite identification: 1, identified compound by 1- and 2-dimensional NMR and other spectral data, such as IR, UV, etc.; 2, putatively annotated compound based on spectral similarity with public/commercial spectral libraries; 3, putatively annotated compound class based upon spectral similarity to known chemical class; and 4, unknown compounds.

### Data availability.

Genome antiSMASH annotation and analyses can be accessed at https://zenodo.org/record/4592481. All transcriptomic data sets used in this study are accessible at the NCBI Gene Expression Omnibus under accession number GSE106136. Feature-based molecular networking on GNPS can be accessed at https://gnps.ucsd.edu/ProteoSAFe/status.jsp?task=36c108371b1f41439b102a8ab75f1fce. The fungal isolates Zt05 and Zt10 were deposited at the CBS Collection of the Westerdijk Fungal Biodiversity Institute under the accession numbers CBS148507 and CBS148508, respectively. The accession number of isolate IPO323 is CBC115943.
