# Supplementary material for: Differential Regulation and Production of Secondary Metabolites among Isolates of the Fungal Wheat Pathogen Zymoseptoria tritici
Source: Appl Environ Microbiol. 2022 Mar 22;88(6):e02296-21. doi: 10.1128/aem.02296-21 (PMC8939313; doi:10.1128/aem.02296-21)
Supplement: Supplemental file 1 — Supplemental table list, Fig. S1 to S7, and supplemental information. Download aem.02296-21-s0006.pdf, PDF file, 6.6 MB [file aem.02296-21-s0006.pdf]

Title:

Differential regulation and production of secondary metabolites among isolates of the fungal wheat pathogen *Zymoseptoria tritici*

Authors:

M. Amine Hassani, Ernest Oppong-Danquah, Alice Feurtey, Deniz Tasdemir, Eva H. Stukenbrock

Supplementary Information

Supplementary file 1: Bioinformatic pipeline of the transcriptomic analyses

Table S1: Predicted BGCs from *Zymoseptoria tritici* isolate Zt05

Table S2: Predicted BGCs from *Zymoseptoria tritici* isolate IPO323

Table S3: Predicted BGCs from *Zymoseptoria tritici* isolate Zt10

Table S4: List of Biosynthetic gene family membership

Table S5: Summary statistics of RNAseq read mapping

Table S6: Differential gene expression *in planta*

Table S7: Transcripts per million read (TPM) *in planta*

Table S8: Detailed annotation of genes in the predicted ABA cluster

Table S9: Putative identification of metabolites in all *Zymoseptoria tritici* isolate

Table S10: Differential gene expression of IPO323 and kmt6 mutant during *in vitro* growth

Figure S1. Expressed and non-detected genes during the course of infection. Bar plot representing the number and percentages of expressed genes and genes with no detectable expression in the three strains of *Z. tritici*. Genes are separated in two categories: the genes predicted to belong to a BGC and all other

genes. Numbers and proportions are given per infection stage.

Figure S2. Transcriptomic profile of core biosynthetic genes during infection. A dotted line represents change in expression level that is not significant whereas significant differences are represented by a full line. The line plots are labeled with the orthogroup number (written as: OGXXXX, X refers to a number) as well as with an identifier for the BGF (BGFXX, X refers to a number) to allow identification of core biosynthetic genes belonging the same cluster. In yellow are highlighted the BGF with some similarity to functionally characterized clusters of other species. The predicted products are as follow: BGF11 – Ustiloxin B; BGF17 – Echinocandin; BGF20 – Abscisic acid; BGF23 – Melanin; BGF28 – Fusaridione; BGF29 – Gliotoxin; BGF5 – Betaenone.

Figure S3. Feature based molecular network (FBMN) of IPO323 and ΔKmt6 mutant. FBMN generated from the Global Natural Product Social MN platform of crude organic extracts from IPO323 and Δkmt6 mutant of *Z. tritici*. Nodes represent molecular ions detected in the crude extracts with colour coding indicative of the relative abundance in each *Z. tritici* strain (red-IPO323 and green-Δkmt6 mutant). Some annotated compounds are displayed with their chemical classes: polyketide (PK), nonribosomal peptide (NRP), terpene (TER) and fatty acid (FA). Other annotations are displayed in Table S9.

Figure S4. GNPS MS/MS mirror plot of experimental and library product ions of phosphatidylcholine annotation. Comparison of the MS/MS spectrum of phosphotidylcholine from the GNPS database (green) to the measured MS/MS spectrum of the annotated node (black).

Figure S5. GNPS MS/MS mirror plot of experimental and library product ions of the diketopiperazine cyclo (Leu-Pro) annotation. Comparison of the MS/MS spectrum of cyclo (Leu-Pro) from the GNPS database (green) to the measured MS/MS spectrum of the annotated node (black).

Figure S6. GNPS MS/MS mirror plot of experimental and library product ions of lumichrome annotation. Comparison of the MS/MS spectrum of lumichrome from the GNPS database (green) to the measured MS/MS spectrum of the annotated node (black).

Figure S7. Comparison of the ^{1}H NMR spectra of IPO323 extract and ABA. ^{1}H NMR spectra of the IPO323 EtOAc extract (Bruker Avance III 500 MHz spectrometer, CD_3OD, up) and of ABA (CD_3OD, down, reference spectrum obtained from the Biological Magnetic Resonance Data Bank (BMRB), https://bmrb.io/).

Figure S1

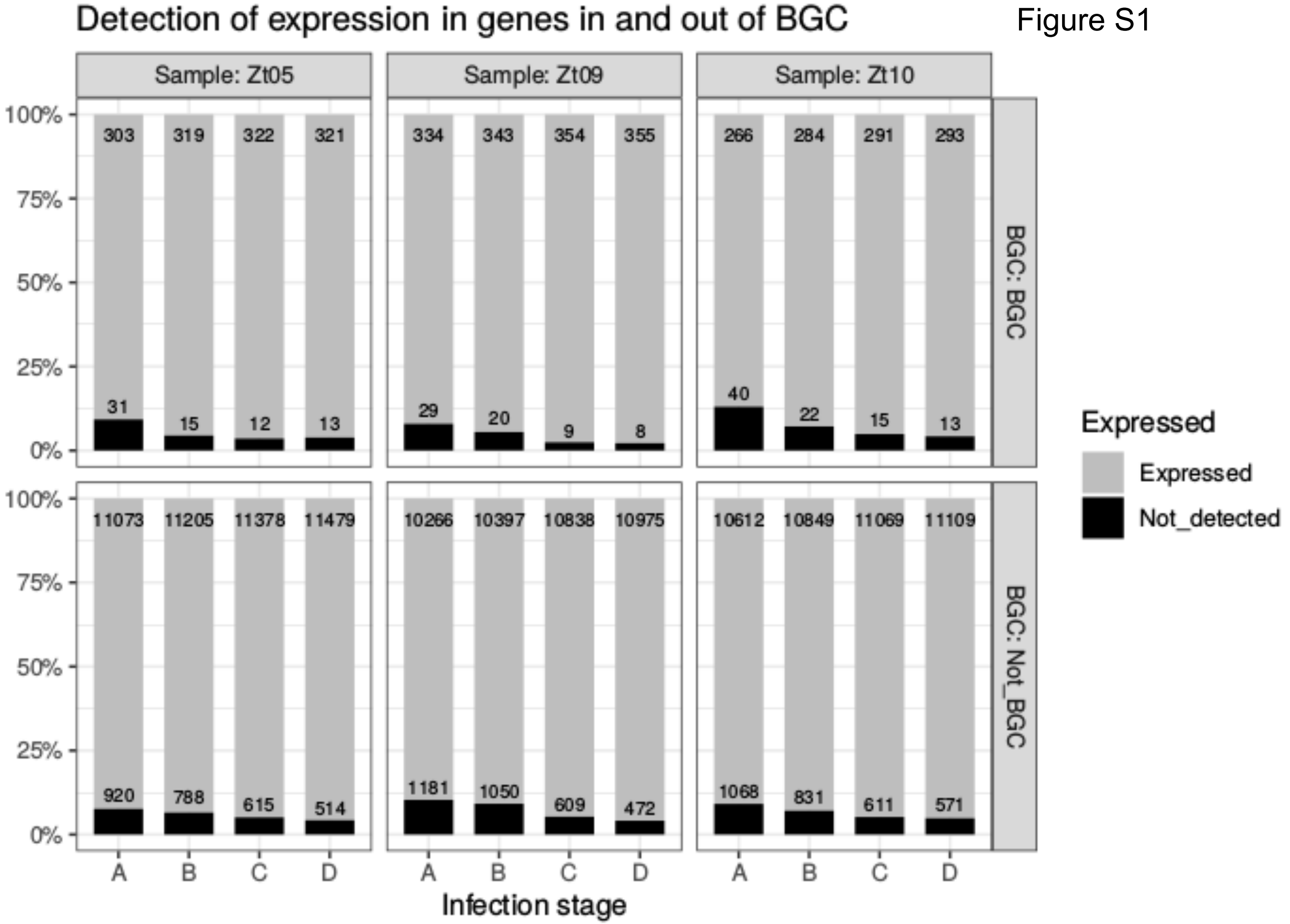
Detection of expression in genes in and out of BGC
Sample: Zt05
Sample: Zt09
Sample: Zt10
BGC: BGC
BGC: Not_BGC
100%
75%
50%
25%
0%
303 319 322 321
31 15 12 13
334 343 354 355
29 20 9 8
266 284 291 293
40 22 15 13
11073 11205 11378 11479
920 788 615 514
10266 10397 10838 10975
1181 1050 609 472
10612 10849 11069 11109
1068 831 611 571
A B C D
Infection stage
Expressed
Expressed
Not_detected

Figure S2

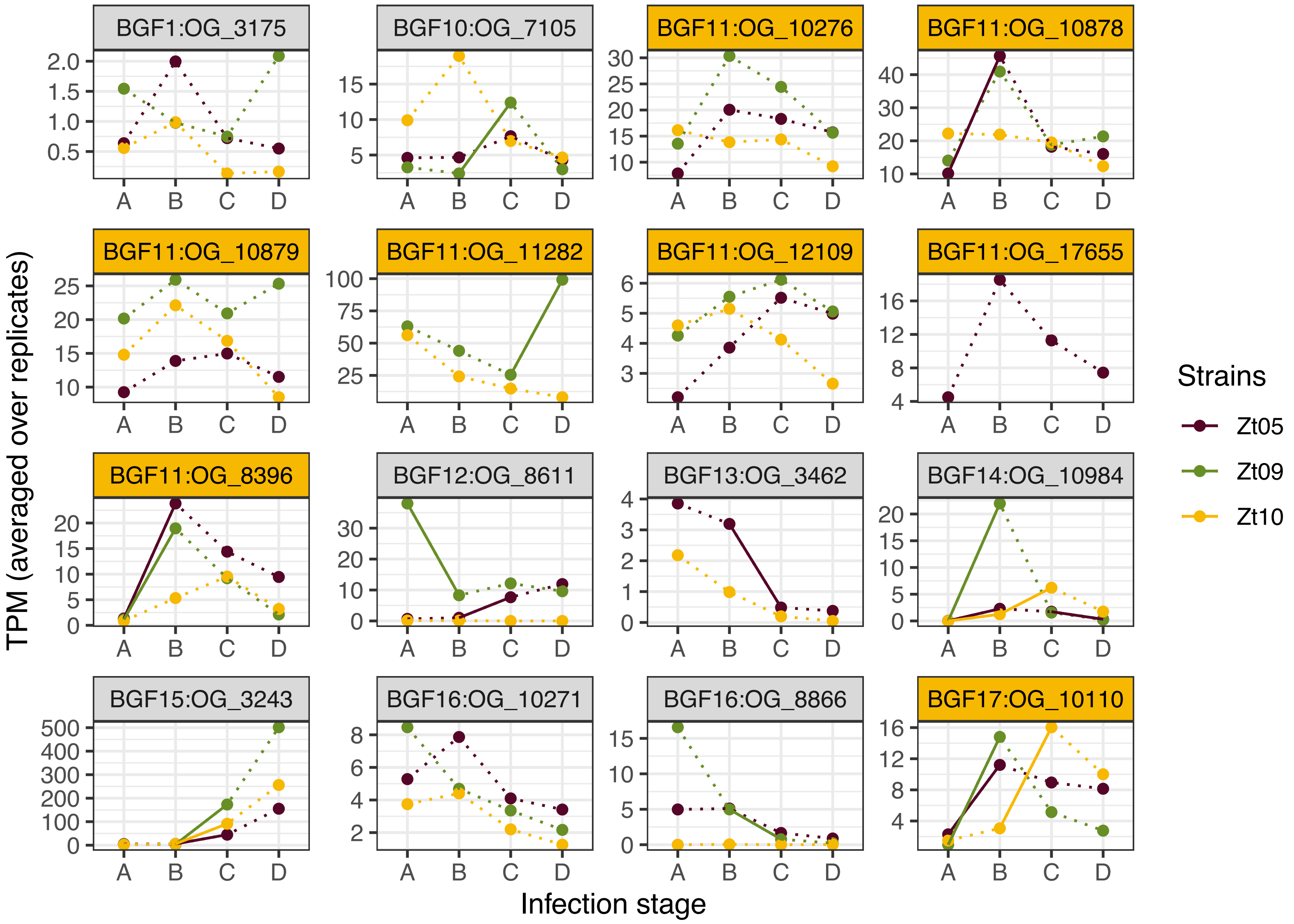
BGF1:OG_3175
BGF10:OG_7105
BGF11:OG_10276
BGF11:OG_10878
BGF11:OG_10879
BGF11:OG_11282
BGF11:OG_12109
BGF11:OG_17655
BGF11:OG_8396
BGF12:OG_8611
BGF13:OG_3462
BGF14:OG_10984
BGF15:OG_3243
BGF16:OG_10271
BGF16:OG_8866
BGF17:OG_10110
TPM (averaged over replicates)
Infection stage
A B C D
Strains
Zt05
Zt09
Zt10

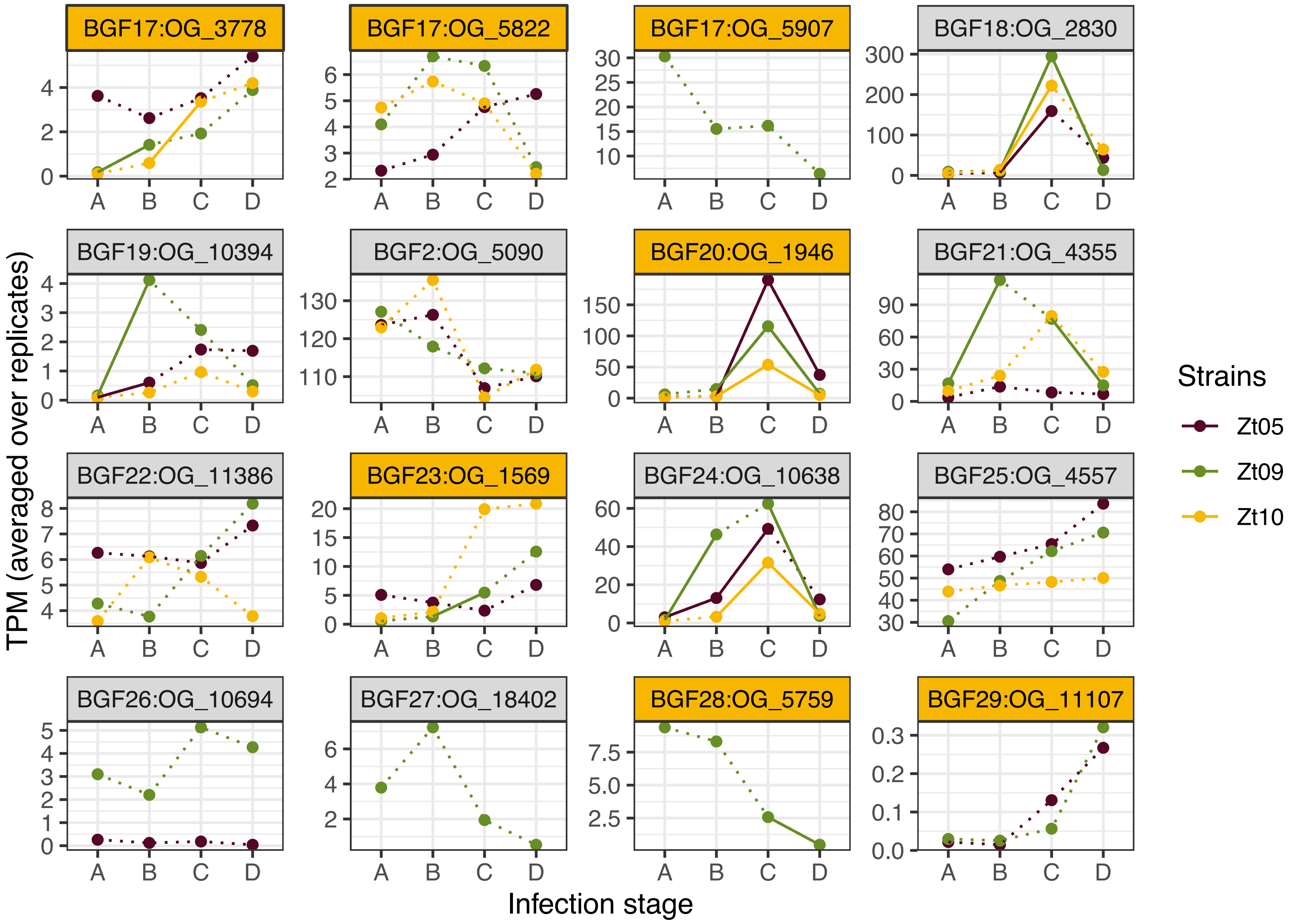
BGF17:OG_3778
BGF17:OG_5822
BGF17:OG_5907
BGF18:OG_2830
BGF19:OG_10394
BGF2:OG_5090
BGF20:OG_1946
BGF21:OG_4355
BGF22:OG_11386
BGF23:OG_1569
BGF24:OG_10638
BGF25:OG_4557
BGF26:OG_10694
BGF27:OG_18402
BGF28:OG_5759
BGF29:OG_11107
TPM (averaged over replicates)
Infection stage
Strains
Zt05
Zt09
Zt10

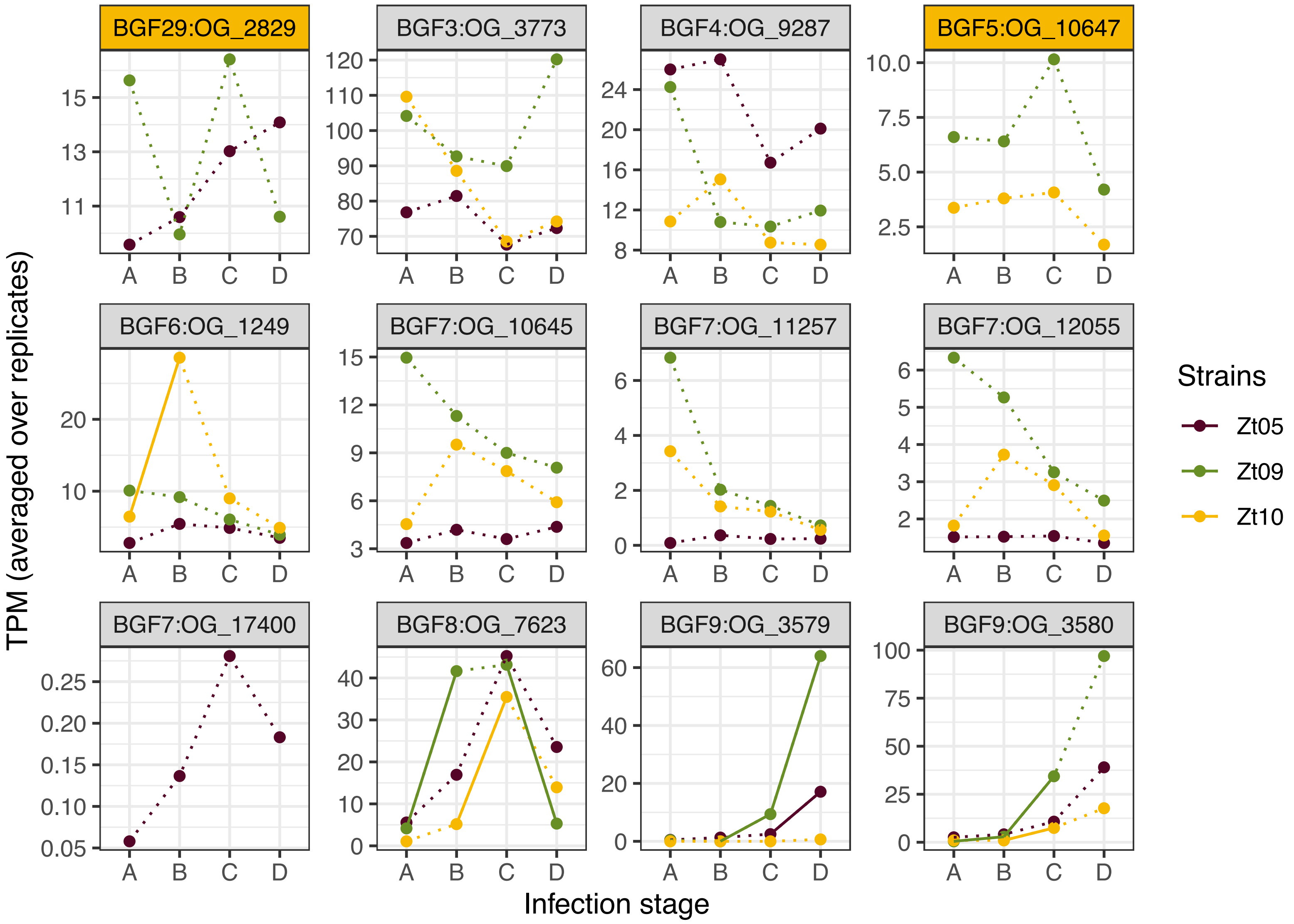
BGF29:OG_2829
BGF3:OG_3773
BGF4:OG_9287
BGF5:OG_10647
BGF6:OG_1249
BGF7:OG_10645
BGF7:OG_11257
BGF7:OG_12055
BGF7:OG_17400
BGF8:OG_7623
BGF9:OG_3579
BGF9:OG_3580
TPM (averaged over replicates)
Infection stage
Strains
Zt05
Zt09
Zt10

IPO323_ ΔKmt FBMN

Figure S3

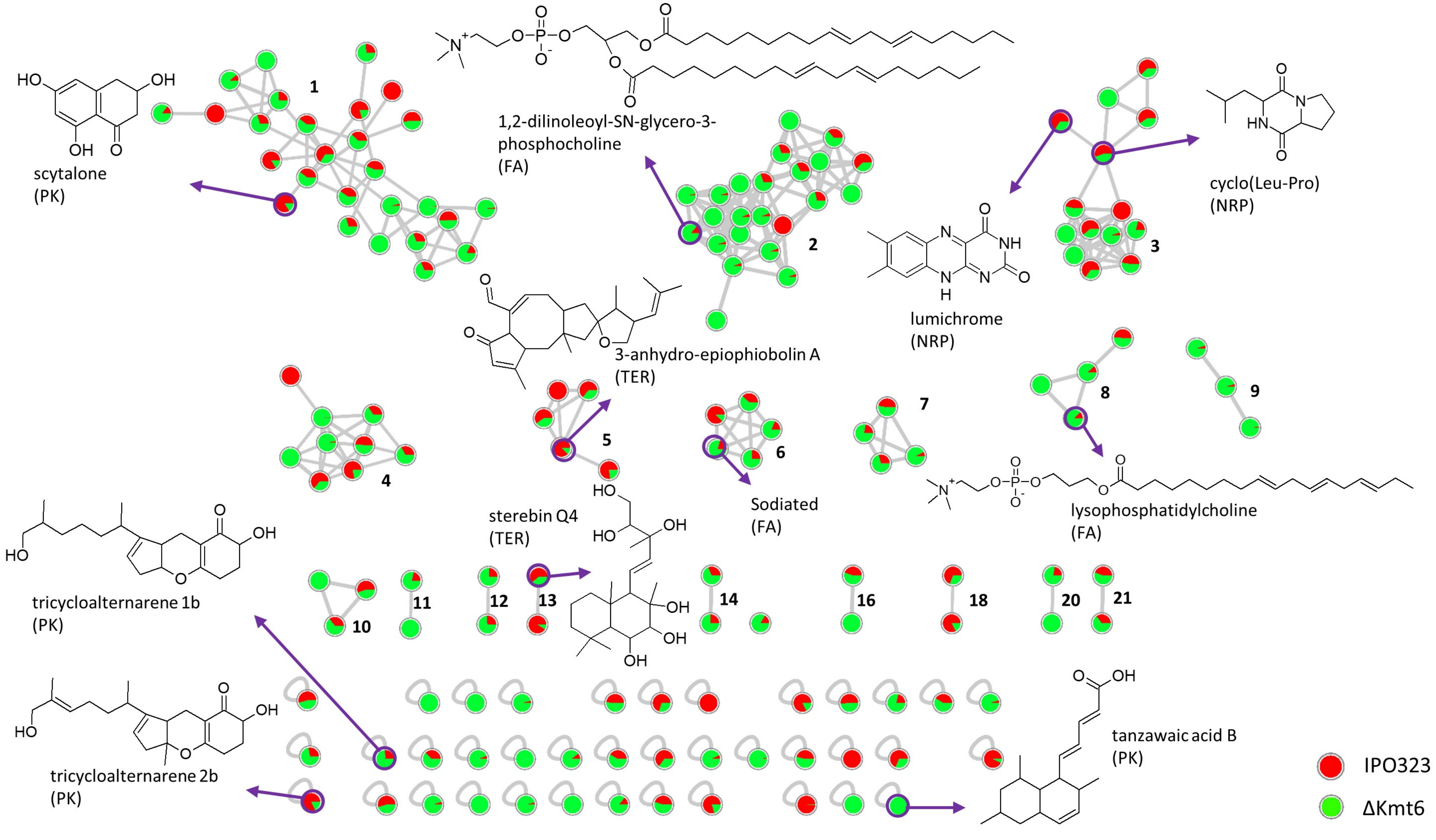

GNPS MS/MS mirror plot phosphocholine

Figure S4

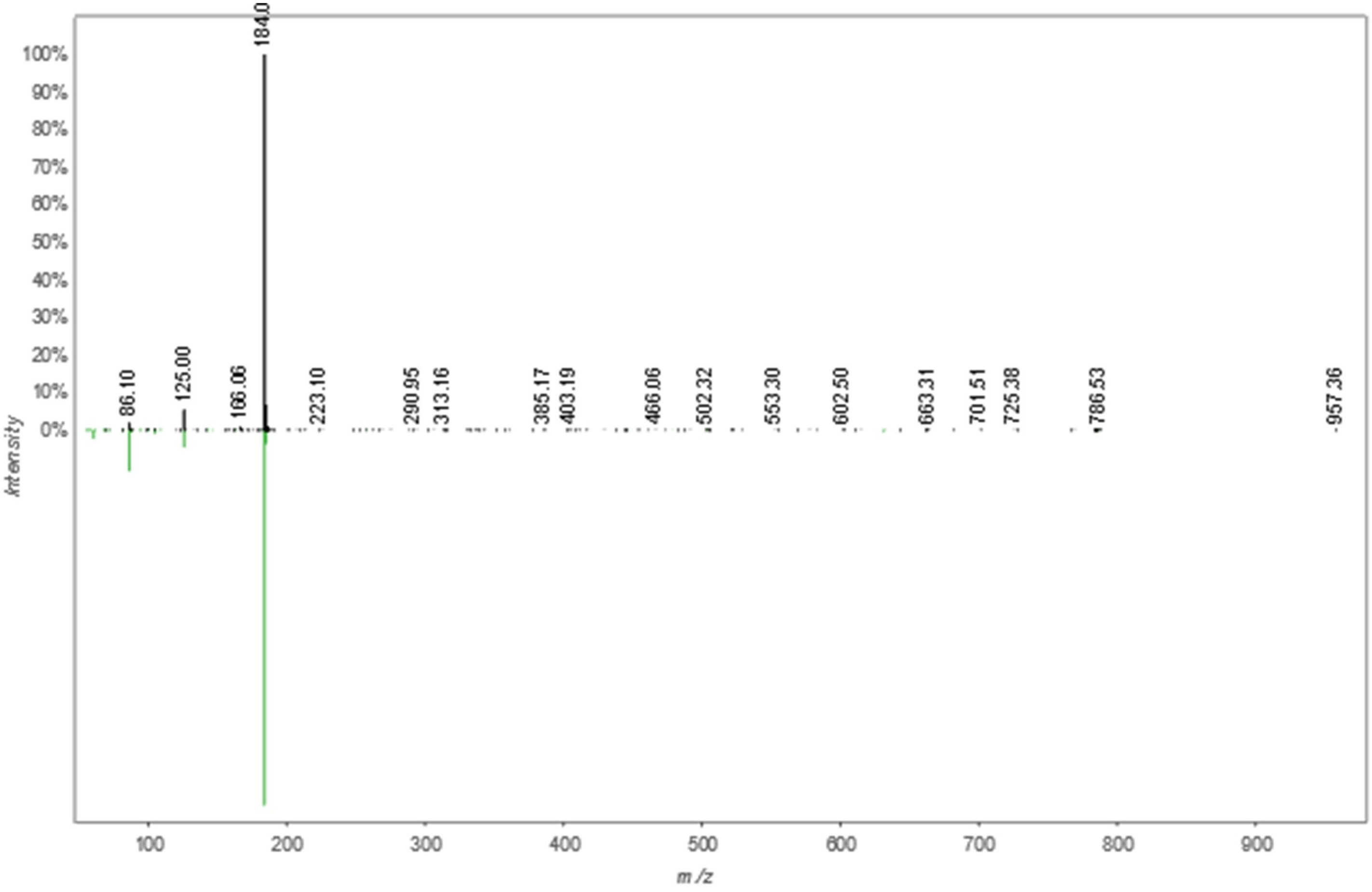

GNPS MS/MS mirror plot of cyclo (Leu-Pro)

Figure S5

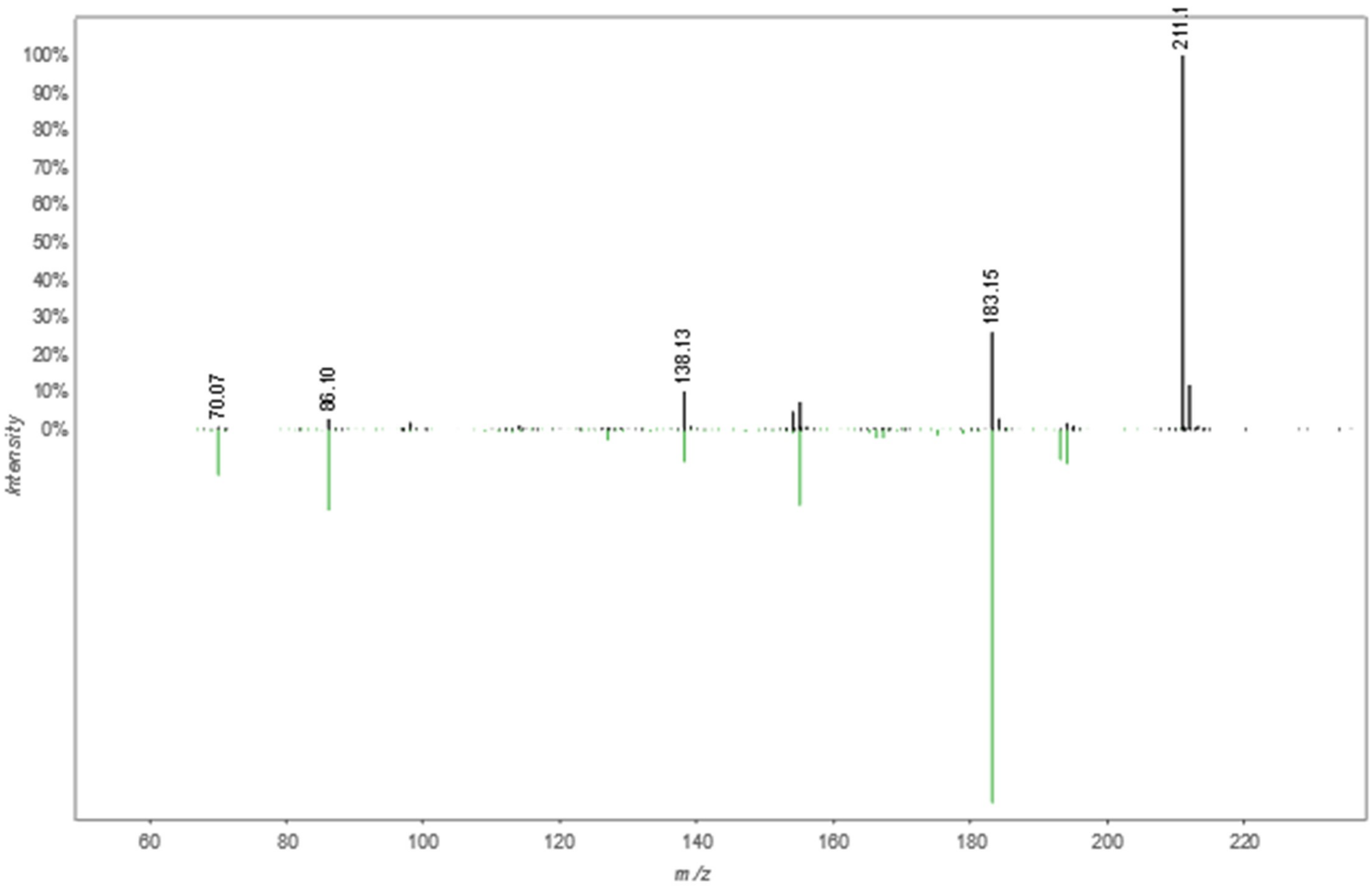

GNPS MS/MS mirror plot lumichrome

Figure S6

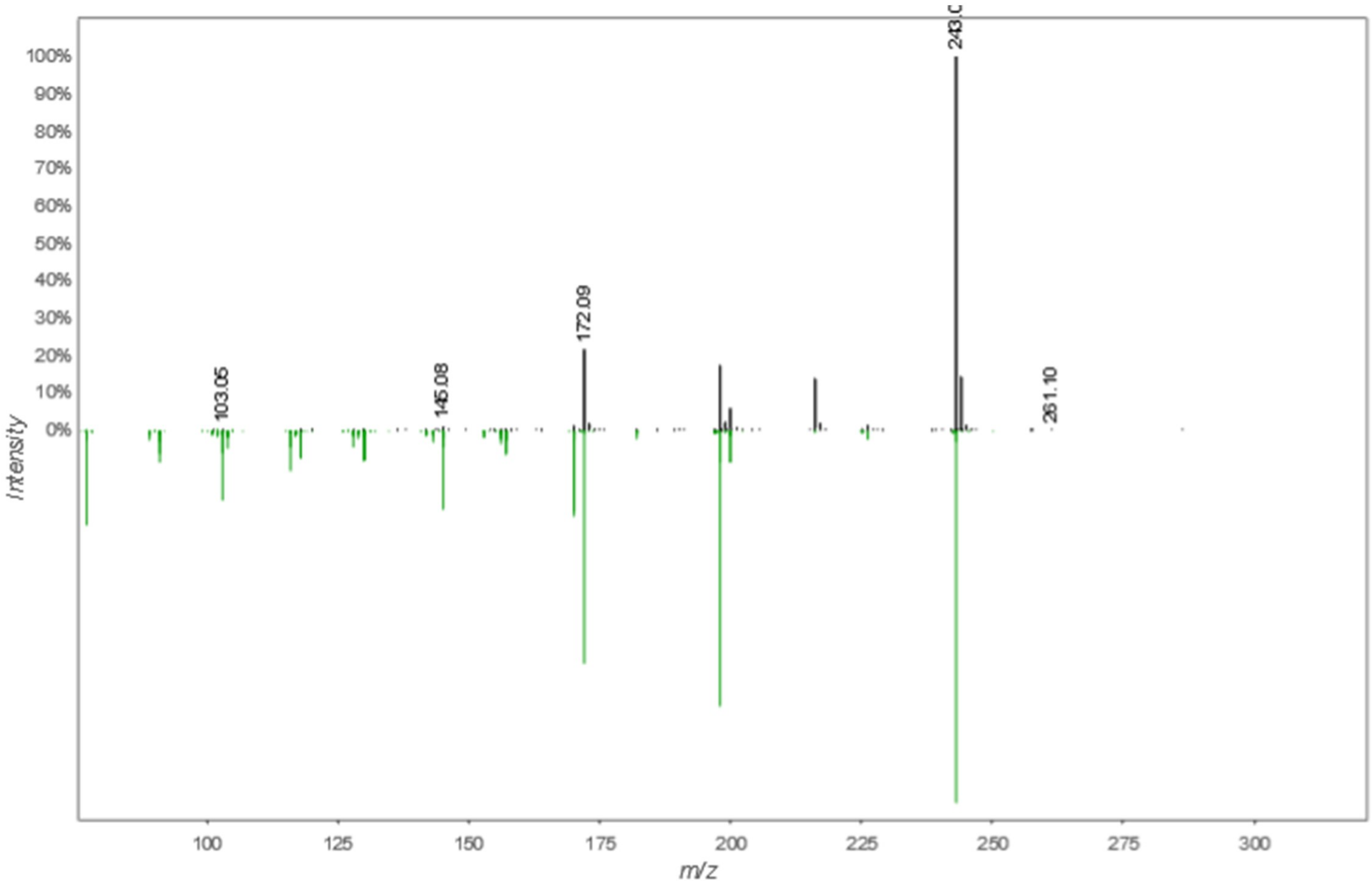

Comparing the ^{1}H NMR spectra of IPO323 extract and ABA

Figure S7

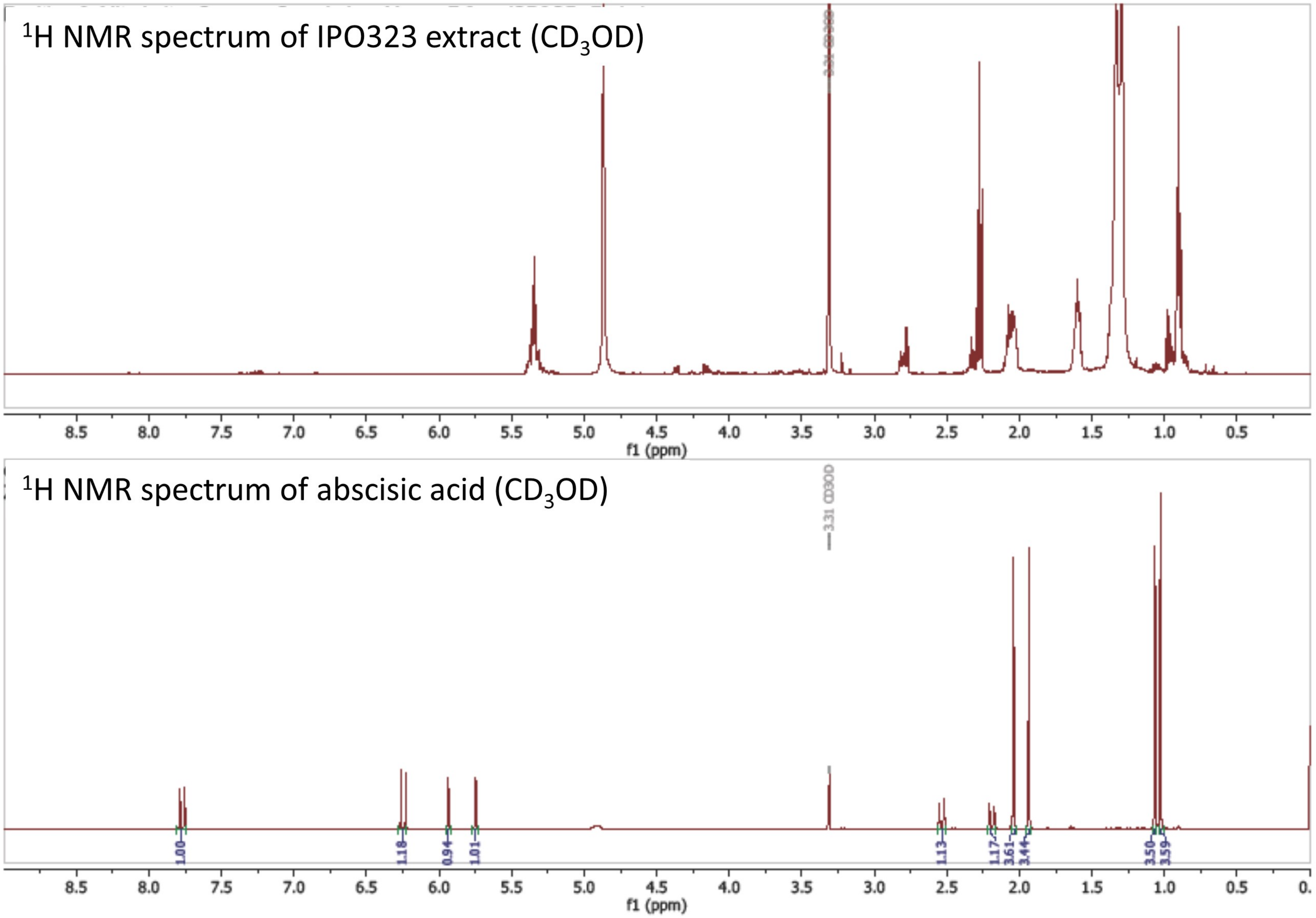

Code

Diversity in secondary metabolite gene clusters and metabolite production among isolates of the fungal wheat pathogen Zymoseptoria tritici

Supplementary Text

Transcriptomic analyses

Hide

```
#Loading relevant packages
library(tidyverse)
library(GenomicFeatures)
library(DESeq2)
library(RColorBrewer)
library(pheatmap)
library(cowplot)
library(kableExtra)
library(gggenes)
library(gridExtra)

#Setting up some default chunk options
knitr::opts_chunk$set(message = F)
knitr::opts_chunk$set(warning = F)
knitr::opts_chunk$set(results = T)

#These are the main directories
work_dir="/Users/feurtey/Documents/Postdoc_Eva/Projects/BGC_Amine/"
BCG_dir=paste0(work_dir, "0_New_BGC/")
RC_dir = paste0(work_dir, "1_Read_counts/")
published_dir = "/Users/feurtey/Documents/Postdoc_Eva/Manuscripts/Alice_Cecile_Comparative_genomics/Data_for_pu
blication/"
to_publish_dir = "/Users/feurtey/Documents/Postdoc_Eva/Manuscripts/Amine_BGC/"


#Some variables and colors
strain_list = c("Zt09", "Zt05", "Zt10")
strain_colors = c("#550527", "#688E26", "#F7B801")
names(strain_colors) = levels(factor(strain_list))
scale_color_strains = ggplot2::scale_colour_manual(name = "Strains", values = strain_colors)
```

Data preparation

Alignment of the RNAseq reads and read counts

I have used published transcriptomic datasets from Haueisen et al. 2019 (https://doi.org/10.1002/ece3.4724) for the *in planta* data from the three strains and from Möller et al. 2019 (https://doi.org/10.1371/journal.pgen.1008093) for the *in vitro* data for the wild reference strains and its kmt6 mutant. The trimming was done with Trimmomatic v0.38, the alignment to each genome assembly (IPO323, Zt05 and Zt10) with HISAT2 version 2.2.1 and the read counting with HTseq v0.11.2. And example script can be found here, but it was run on the Wallace cluster (MPI for Evoutionary Biology) in November and December 2020.

Hide

```
work_dir="/home/feurtey/Various/BGC_Amine/Zt09/"
unfiltered_dir="0-Untrimmed_reads/"
filtered_dir="1-Trimmed_reads/"
aligned_dir="2-Alignments/"
counts_dir="3-Read_counts/"

cd ${work_dir}${unfiltered_dir}
for fq_file in S024*fastq* ; do
    echo ${fq_file}
    echo ${work_dir}${unfiltered_dir}${fq_file%fastq*}adapt.qual30.minlen30.fastq.gz

    fastqc ${fq_file}

    java -jar /data/biosoftware/Trimmomatic/Trimmomatic-0.38/trimmomatic-0.38.jar \
     SE \
     ${fq_file} \
     ${work_dir}${filtered_dir}${fq_file%fastq*}adapt.qual30.minlen30.fastq.gz \
     ILLUMINACLIP:${work_dir}${filtered_dir}adapters.txt:2:30:10 \
     LEADING:30 TRAILING:30 MINLEN:30

    fastqc ${work_dir}${filtered_dir}${fq_file%fastq*}adapt.qual30.minlen30.fastq.gz
done

mkdir ${work_dir}${aligned_dir}By_strand/

hisat2-build ${work_dir}${aligned_dir}Zt09.fasta ${work_dir}${aligned_dir}Zt09

cd ${work_dir}${filtered_dir}

for fq_file in *adapt.qual30.minlen30.fastq.gz; do
    aln_file=${fq_file%.adapt.qual30.minlen30.fastq.gz}

    hisat2 \
     -x ${work_dir}${aligned_dir}Zt09 \
     -U $fq_file \
     -S ${work_dir}${aligned_dir}${aln_file}.sam \
     --un ${work_dir}${aligned_dir}${aln_file}_unaligned_reads.fastq \
     --no-unal --met-stderr --phred33 \
     --rna-strandness R \
     --min-intronlen 20 --max-intronlen 15000 \
     --summary-file ${work_dir}${aligned_dir}${aln_file}_summary.txt \
     --threads 4

     samtools flagstat ${work_dir}${aligned_dir}${aln_file}.sam \
      > ${work_dir}${aligned_dir}${aln_file}_flagstat.txt

     samtools view -b ${work_dir}${aligned_dir}${aln_file}.sam > ${work_dir}${aligned_dir}${aln_file}.bam

done

module load python/2.7.13
cd ${work_dir}${aligned_dir}
for sam_file in *sam ; do
    echo ${sam_file}
    prefix=${sam_file%.sam}

    htseq-count -m union --type=gene --idattr=Name --stranded=reverse \
       ${work_dir}${aligned_dir}${prefix}.sam \
       ${work_dir}Ztritici_annotation_Grandaubert_wUTR.gff \
       > ${work_dir}${counts_dir}${prefix}_read_counts.txt

done
```

In gene annotations, there often are genes which are part of transposons such as reverse transcriptases or transposases. Although these are real genes, they are not necessarily relevant for most gene analysis. I will remove all genes which have been predicted to have either function from Feurtey et al. 2020 (https://doi.org/10.1186/s12864-020-06871-w) (by emapper).

Hide

```
#Identify "genes" which actually correspond to transposases etc
echo "reverse transc" > temp.txt
echo "transpos" >> temp.txt

grep -f temp.txt /Users/feurtey/Documents/Postdoc_Eva/Manuscripts/Alice_Cecile_Comparative_genomics/Data_for_pu
blication/Annotations_emapper_2018_genomes_for_publication.tab --ignore-case | cut -f 2 > Genes_annotated_as_tr
ansposons.txt

#Exclude them from the counts
for fileName in 1_Read_counts/In_vitro/S*_read_counts.txt ; do grep -f Genes_annotated_as_transposons.txt ${fil
eName} -v > ${fileName%.txt}_woTEgenes.txt ; grep -f Genes_annotated_as_transposons.txt ${fileName} -c ; done

for fileName in 1_Read_counts/In_planta/S*_read_counts.txt ; do grep -f Genes_annotated_as_transposons.txt ${fi
leName} -v > ${fileName%.txt}_woTEgenes.txt ; grep -f Genes_annotated_as_transposons.txt ${fileName} -c ; done
```

This resulted in **59** genes being filtered out for Zt05, **29** for Zt09 and **5** for Zt10.

Metadata : BGC and other annotations

Amine has run antiSMASH v5.2 (check this with him) using the our last gff files and assemblies (https://doi.org/10.5281/zenodo.3568212). I parse the downloaded results here from the genebank files for each cluster (ex: chr_1.region001.gbk) and import the gene names and gene kind (as defined by antiSMASH v.5.2).

Hide

```
#This code was run in a folder containing the downloaded and unzipped results from the web-based antiSMASH.
#It parses the genebank files for each cluster (ex: chr_1.region001.gbk) to get the gene names and the "gene ki
nd".

echo "gene=" > temp.txt
echo "gene_kind=" >> temp.txt

grep -f temp.txt */*region*.gbk | awk 'BEGIN {FS="/"; OFS = "\t"} {print $3, $2}' | sed 's/://' | tr "\n" "\t"
 | gsed "s/gene=/\n/g" | \
>   cut -f 1,2,3 |  sed 's/"//g' | gsed 's/gene_kind=//g' > list_genes_clusters.txt
```

Hide

```
#Import BGC from Amine new analyses
BGC_genes = read_tsv(paste0(BCG_dir, "list_genes_clusters.txt") ,
         col_names = c("Gene", "Cluster_Amine", "Gene_kind"))
BGF = read_tsv(paste0(BCG_dir, "BGF_table_from_Jaccard.txt"))
```

In order to calculate the Transcripts Per kilobase Million (TPM), we need to have the exonic gene length (sum of exon length per gene). I calculate this for all genes and add this information to the annotations from Feurtey et al. 2020 and the new BGC annotation.

Hide

```
# Import GFF for all genes, get the exons and sum
txdb_all <- makeTxDbFromGFF("/Users/feurtey/Documents/Postdoc_Eva/Projects/BGC_Amine/Ztritici_annotation_Granda
ubert_wUTR.gff",format="auto")
exons.list.per.gene <- exonsBy(txdb_all,by="gene")
gene_details <- as_tibble(data.frame(Exonic_gene_size = sum(width(reduce(exons.list.per.gene)))),
                        rownames = "Gene")
#Add Zt05
txdb_all <- makeTxDbFromGFF(paste0(published_dir, "Zt05_2019_for_publication.gff3"),format="auto")
exons.list.per.gene <- exonsBy(txdb_all,by="gene")
gene_details <- bind_rows(gene_details,
                        as_tibble(data.frame(Exonic_gene_size = sum(width(reduce(exons.list.per.gene)))),
                                  rownames = "Gene"))
#Add Zt10
txdb_all <- makeTxDbFromGFF(paste0(published_dir, "Zt10_2019_for_publication.gff3"),format="auto")
exons.list.per.gene <- exonsBy(txdb_all,by="gene")
gene_details <- bind_rows(gene_details,
                        as_tibble(data.frame(Exonic_gene_size = sum(width(reduce(exons.list.per.gene)))),
                                  rownames = "Gene"))
#Add info from our paper
gene_details = inner_join(gene_details,
                          read_tsv(paste0(published_dir, "Annotations_2018_genomes_for_publication.tab")),
                          by = c("Gene" = "Protein_ID")) %>%
  dplyr::select("Gene", "Exonic_gene_size", "Contig", "Start", "End", "Strand",
                "Secretion", "Transmembrane", "Cell_location", "Effector", "CAZyme", "CAZyme_family",
                "Sample", "Orthogroup", "is_para", "Common_Zymo", "Common_Zt")


#Let's add the data about the BGF created by Amine
temp = BGF %>%
  pivot_longer(-BGF, names_to = "X1", values_to = "Cluster_Amine_underscore") %>%
  dplyr::select(-X1) %>%
  filter(complete.cases(.))


gene_details = full_join(gene_details, BGC_genes) %>%
  mutate(Cluster_Amine_underscore = gsub('.gbk', '', Cluster_Amine)) %>%
  mutate(Cluster_Amine_underscore = gsub('\\.', '_', Cluster_Amine_underscore)) %>%
  unite(Sample, Cluster_Amine_underscore, sep = "_", col = "Cluster_Amine_underscore", remove = FALSE) %>%
  mutate(Cluster_Amine_underscore = ifelse(is.na(Cluster_Amine), NA, Cluster_Amine_underscore)) %>%
  full_join(., temp,
            by = "Cluster_Amine_underscore")



#Write a summary table for Amine
gene_details %>%
  dplyr::select(Sample, Gene, Contig, Start, End, Orthogroup, Cluster_Amine, Gene_kind, Cluster_Amine_underscor
e) %>%
  filter(!is.na(Cluster_Amine)) %>%
  write_tsv(paste0(work_dir, "Correspondance_Table_BGC_genes_orthogroup.txt"))


#List of the clusters that have a known function
known_BGC = read_tsv(paste0(work_dir, "BGC_with_functions.txt")) %>%
  pivot_longer(-c(Cluster_Function, Biosynthetic_gene_nb), names_to = "Sample", values_to = "Gene") %>%
  filter(complete.cases(.)) %>%
  full_join(., gene_details) %>%
  dplyr::select(Cluster_Amine, Cluster_Function, Sample) %>%
  unique()%>%
  filter(!is.na(Cluster_Function))

#To add the biosynthetic gene number
temp = read_tsv(paste0(work_dir, "BGC_with_functions.txt")) %>%
  pivot_longer(-c(Cluster_Function, Biosynthetic_gene_nb), names_to = "Sample", values_to = "Gene") %>%
  filter(complete.cases(.))
```

```
gene_details =full_join(gene_details, known_BGC, by = c("Cluster_Amine", "Sample")) %>%
  full_join(., temp)
```

Expression *in planta* between strains and infection stages

Hide

```
## Reading the data

suffix_RC="read_counts_woTEgenes"

#Metadata
sampleCondition_inplanta <- c("in_planta_A", "in_planta_A", "in_planta_B", "in_planta_B",
                           "in_planta_C", "in_planta_C", "in_planta_D", "in_planta_D")
sampleCondition_invitro <- c("in_vitro_wild", "in_vitro_wild", "in_vitro_kmt6", "in_vitro_kmt6")

condition_list <- c(sampleCondition_invitro, rep(sampleCondition_inplanta, 3))

#All files
sampleCondition_inplanta <- c("in_planta_A", "in_planta_A", "in_planta_B", "in_planta_B",
                           "in_planta_C", "in_planta_C", "in_planta_D", "in_planta_D")
condition_list <- c(sampleCondition_invitro, rep(sampleCondition_inplanta, 3))
strain_list <- c(rep("Zt09", 4), rep("Zt05", 8), rep("Zt09", 8), rep("Zt10", 8))
#sampleTable = bind_rows(sampleTable_invitro, sampleTable_inplanta)
Paths = c(paste0(paste0(RC_dir, "In_vitro/"),
                 grep(suffix_RC, list.files(paste0(RC_dir, "In_vitro")), value=TRUE)),
          paste0(paste0(RC_dir, "In_planta/"),
                 grep(suffix_RC, list.files(paste0(RC_dir, "In_planta")), value=TRUE)))
```

TPM

Let’s firt import the genes from the 3 strains and load the corresponding annotation from Feurtey et al. 2020. Then I estimate the TPM. I chose this value because it allows comparison between different samples (https://rna-seqblog.com/rpkm-fpkm-and-tpm-clearly-explained/).

Hide

```
data <- tibble(fileName = Paths) %>%
  mutate(Condition = condition_list,
         strain = strain_list) %>%
  mutate(file_contents = map(fileName,
           ~ read_tsv(file.path(.),
                      col_names = c("Gene", "Count")))) %>%
  unnest(cols = c(file_contents)) %>%
  left_join(., gene_details) %>%
  mutate(BGC = ifelse(!is.na(Cluster_Amine), "BGC", "Not_BGC")) %>%
  mutate(RPK = Count/(Exonic_gene_size/1000)) %>%
  filter(!grepl('^_', Gene))

scaling_factor = group_by(data, fileName) %>%
  summarize(scaling_factor = sum(RPK)/1000000)

average_TPM = left_join(data, scaling_factor) %>%
  mutate(TPM = RPK/scaling_factor) %>%
  group_by(Gene, Sample, Orthogroup, Contig, Secretion, Effector,
           Cluster_Amine, Cluster_Amine_underscore, Gene_kind, Condition, BGC, BGF,
           Cluster_Function, Biosynthetic_gene_nb) %>%
  summarize(av_TPM = mean(TPM), av_Count = mean(Count)) %>%
  mutate(Expressed = ifelse(av_Count >= 1, "Expressed", "Not_detected"))


#Writing the table for supplementary material
average_TPM  %>%
  ungroup() %>%
  dplyr::select(Gene, Sample, Orthogroup,
                Cluster_Amine, Cluster_Function, BGF, Gene_kind,
                Condition, av_TPM, av_Count) %>%
  rename(av_TPM = "TPM (averaged over replicates)",
         av_Count = "Read count (averaged over replicates)",
         Cluster_Amine = "BGC") %>%
  write_tsv(paste0(to_publish_dir, "TPM_in_planta.txt"))
```

Once the data is in the memory, I want to see if the genes in BGC are expressed in planta.

Hide

```
brks <- c(0, 0.25, 0.5, 0.75, 1)

temp_all = average_TPM %>%
  filter(BGC != "BGC") %>%
  filter(grepl("planta", Condition)) %>%
  mutate(Condition = gsub("in_planta_", "", Condition)) %>%
  group_by(Sample, Condition, Expressed) %>%
  summarise(count = n()) %>%
  mutate(perc = count/sum(count)) %>%
  mutate(BGC = "Not_BGC")

#Calculating percentages of genes with expression and genes without any detected reads
temp = average_TPM %>%
  filter(BGC == "BGC") %>%
  filter(grepl("planta", Condition)) %>%
  mutate(Condition = gsub("in_planta_", "", Condition)) %>%
  group_by(Sample, Condition, Expressed) %>%
  summarise(count = n()) %>%
  mutate(perc = count/sum(count)) %>%
  mutate(BGC = "BGC") %>%
  bind_rows(., temp_all)

#Bar plot with percentage per strain for both BGC and other genes
plot_1 = temp %>%
  ggplot(aes(x = factor(Condition), y = perc, fill = factor(Expressed))) +
  geom_bar(stat="identity", width = 0.7) +
  ylim(c(0, 1.1))+
  scale_y_continuous(breaks = brks, labels = scales::percent(brks)) +
  labs(x = "Infection stage", y = NULL, fill = "Expressed") +
  theme_bw() +
  labs(title = "Detection of expression in genes in and out of BGC") +
  scale_fill_manual(values = c("grey", "black")) +
  facet_grid(BGC~Sample, labeller=label_both)


#Adding gene numbers to the plot
plot_1 = plot_1 +
  geom_text(data = temp %>%
              filter(Expressed == "Expressed") %>%
              mutate(perc = round(perc, 2)) ,
            aes(y = 0.9, label=count,), vjust = -0.5, size=2.5) +
  geom_text(data = temp %>%
              filter(Expressed == "Not_detected") %>%
              mutate(perc = round(perc, 2)) ,
            aes(label=count,), position=position_dodge(width = 1), vjust = -0.5, size=2.5)

plot_1
```

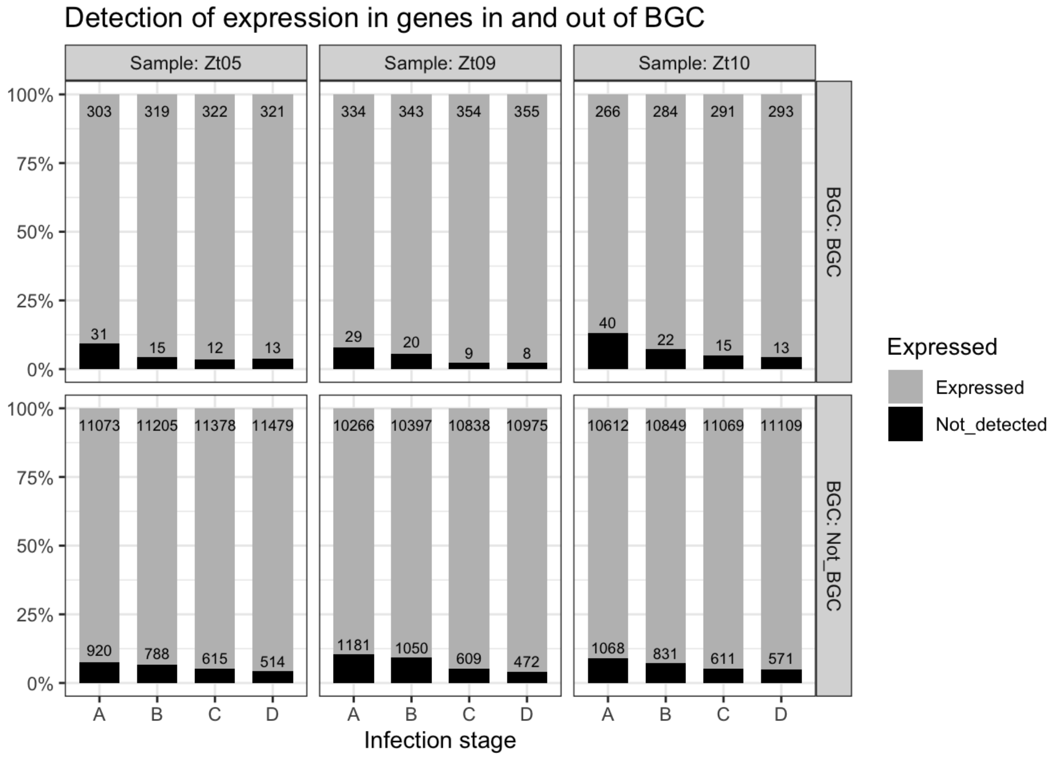

Hide

```
ggsave(paste0(work_dir, "Fig_expressed_genes.pdf"))


#
temp %>% filter(Expressed == "Expressed") %>%
  mutate(perc = round(perc*100, 2)) %>%
  filter(BGC == "BGC") %>%
  dplyr::select(Sample, Condition, perc) %>%
  pivot_wider(names_from = Condition, values_from =perc) %>%
  kbl() %>%
  kable_styling(bootstrap_options = "striped", full_width = F, position = "left")
```

Sample	A	B	C	D
Zt05	90.72	95.51	96.41	96.11
Zt09	92.01	94.49	97.52	97.80
Zt10	86.93	92.81	95.10	95.75

Indeed most are expressed *in planta*. We can see a trend of more gene without detected expression earlier in the infection cycle. However, this trend it also found in the rest of the genes. It is probably due to the fact that the sequencing has not reached saturation in the early stages: genes with low expression are not detected earlier even when some of them are detected later on. I do not think this is significant biologically, but still it could be reported still.

Biosynthetic gene clusters contain different types of genes including one or more main “biosynthetic” gene. Here, I focus on the expression of these main genes *in planta* during the course of the infection.In order to be able to plot all three strains together, I used the previously identified orthogroups. The “title” of each little plot here is the number of the orthogroup. The details of these genes can be found here (https://doi.org/10.5281/zenodo.3568212), in the Annotation_2018 file.

For some clusters, we actually have some ideas of what they can do, thanks to antiSMASH results. So I pulled these out specifically and plotted the expression profile of their core biosynthetic genes across infection stages.

Hide

```
known_BGC_core = average_TPM  %>%
  filter(!is.na(Cluster_Function)) %>%
  filter(Gene_kind == "biosynthetic")


plot_TPM_knownBGC = known_BGC_core %>%
  dplyr::select(Cluster_Function, Biosynthetic_gene_nb, Condition, Sample, av_TPM) %>%
  filter(Condition !=  "in_vitro_kmt6") %>%
  filter(Condition !=  "in_vitro_wild") %>%
  mutate(Condition = gsub("in_planta_", "", Condition)) %>%
    ggplot(aes(x = Condition, y = as.numeric(av_TPM), color = Sample)) +
    geom_point(alpha = 0.8) +
    geom_line(aes(group = Sample), linetype = "dotted") +
    facet_wrap(vars(Cluster_Function,Biosynthetic_gene_nb), scales = "free") +
    theme_bw() +
    scale_color_strains
plot_TPM_knownBGC
```

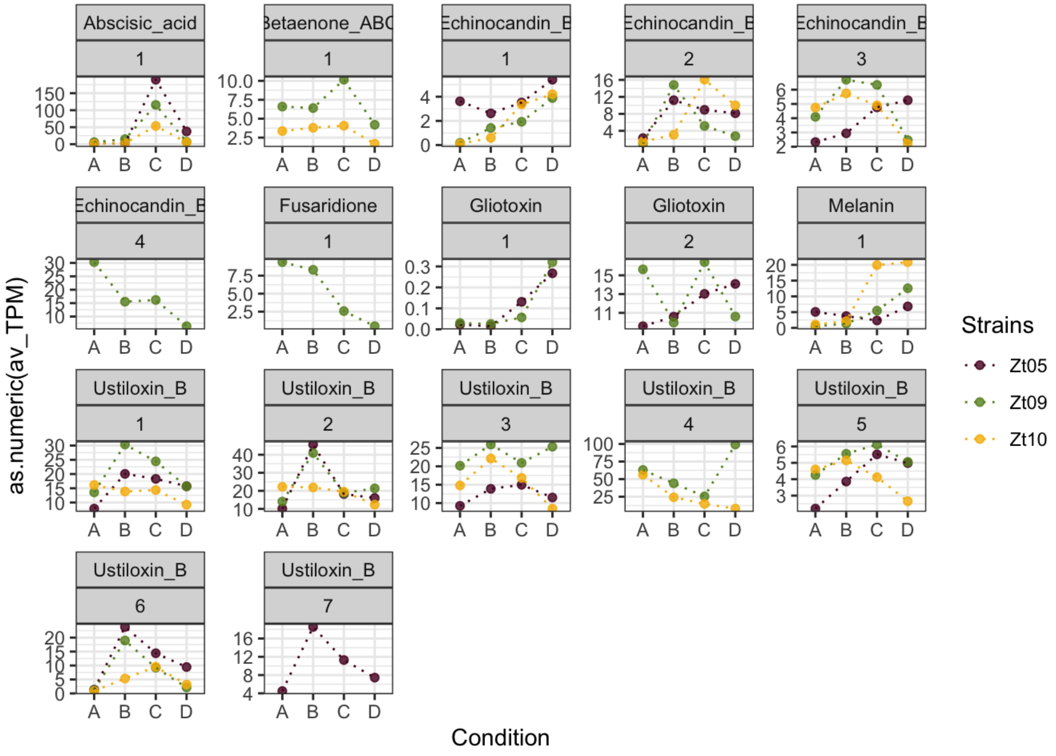

Differential expression with DEseq2

Now that we observed some patterns in the transcription over time we need to decide whether the differences are significant or not. I use DESeq2 for this and the threshold defined by Mareike for her paper on kmt6/wild type, so that the whole paper gives consistent results. Instead of doind an all-against-all analysis with the different infection stages, I try to look for significant differences between “neighbouring” timepoints. So I compare the stage A with the stage B, B with C and C with D.

Hide

```
#Reading the data
all_sampleFiles_inplanta <- grep(suffix_RC,
                    list.files(paste0(RC_dir,"In_planta")),
                    value=TRUE)

list_results=list()
for (sample in c("Zt05", "Zt09", "Zt10")){
sampleFiles_inplanta <- grep(sample, all_sampleFiles_inplanta, value=TRUE)
sampleTable_inplanta <- data.frame(sampleName = sampleFiles_inplanta ,
                          fileName = sampleFiles_inplanta ,
                          condition = sampleCondition_inplanta)
sampleTable_inplanta$condition  <- factor(sampleTable_inplanta$condition )

#Read the data into DESeq2 format
dds_inplanta  <- DESeqDataSetFromHTSeqCount(sampleTable = sampleTable_inplanta ,
                                       directory = paste0(RC_dir,"In_planta"),
                                       design= ~ condition)
dds_inplanta


#Pre-filtering of low values in_vitro
keep <- rowSums(counts(dds_inplanta)) >= 1
dds_inplanta <- dds_inplanta[keep,]

dds_inplanta <- DESeq(dds_inplanta)

resultsNames(dds_inplanta)

#Not all comparisons are relevant, so I select the ones that make sense biologically
res_AB <- results(dds_inplanta, contrast=c("condition","in_planta_A", "in_planta_B"), test="Wald")
res_BC <- results(dds_inplanta, contrast=c("condition","in_planta_B", "in_planta_C"), test="Wald")
res_CD <- results(dds_inplanta, contrast=c("condition","in_planta_C", "in_planta_D"), test="Wald")


#And now I gather all results in one single dataframe for manipulating later
list_results[[sample]] = bind_rows(as_tibble(res_AB, rownames = "Gene") %>%
                   mutate(Comparison = "A_B") %>%
                   mutate(Sample = sample) ,
                 as_tibble(res_BC, rownames = "Gene") %>%
                   mutate(Comparison = "B_C") %>%
                   mutate(Sample = sample)) %>%
  bind_rows(., as_tibble(res_CD, rownames = "Gene") %>%
                   mutate(Comparison = "C_D")) %>%
                   mutate(Sample = sample)  %>%
  mutate(Diff = ifelse(padj <= 0.001,
                     ifelse(abs(log2FoldChange) > 2, "Significant", "Not_significant"),
                     "Not_significant")) %>%
  left_join(., gene_details, by = c("Gene", "Sample")) %>%
  mutate(BGC = ifelse(is.na(Cluster_Amine), "Not_BGC", "BGC"))

}

In_planta_diff_results = bind_rows(list_results)

#Writing the table for supplementary material
In_planta_diff_results  %>%
  dplyr::select(Gene, Sample, Orthogroup,
                Cluster_Amine, Cluster_Function, BGF,
                Gene_kind, Comparison,
                baseMean, log2FoldChange, lfcSE, stat, pvalue, padj) %>%
  rename(Cluster_Amine = "BGC") %>%
  write_tsv(paste0(to_publish_dir, "Differential_expression_in_planta.txt"))
```

Here are the results from the differential expression over all transitions between stages.

Hide

```
#Get overall percentages of genes differentially expressed in at least one of the condition for both BGC and no
n-BGC.
significant = In_planta_diff_results  %>%
  filter(Diff == "Significant") %>%
  dplyr::select(BGC, Comparison, Sample, Gene_kind, Gene) %>%
  unique()
nonsignificant = In_planta_diff_results  %>%
  filter(Diff != "Significant") %>%
  dplyr::select(BGC, Comparison, Sample, Gene_kind, Gene) %>%
  unique()

temp = full_join(as.data.frame(table(significant$BGC, significant$Sample)),
          as.data.frame(table(nonsignificant$BGC, nonsignificant$Sample)),
          by = c("Var1", "Var2")) %>%
   mutate(Percent = (100*Freq.x) /(Freq.x + Freq.y))

p = ggplot(temp, aes(x = Var1, y = Percent, fill = Var1, label = Freq.x)) +
  geom_bar(stat = "identity") +
  geom_text(position = position_dodge(width = .9), vjust = -0.5, size = 3) +
  theme_bw() +
  theme(legend.position = "None") +
  facet_grid (col = vars(Var2)) +
  scale_fill_manual(values = c("#3B5115", "#A0D04E")) +
  labs(y = "Percentage of genes differentially expressed", x = "",
       title = "Percentage of genes differentially expressed",
       subtitle = "in at least one of the comparison between infection stages")

p
```

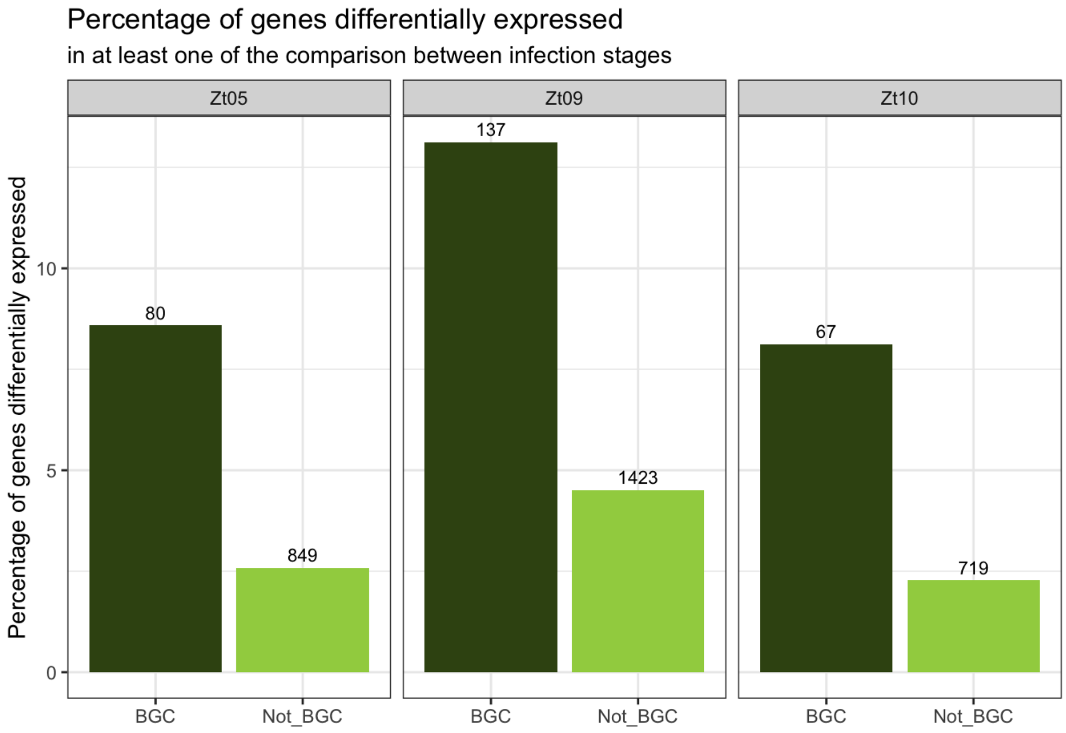

Hide

```
ggsave(paste0(work_dir, "Fig2A.pdf"), width = 4, height = 4)
```

In general, it looks like genes found in BGC have more differential changes in expression across the course of the infection than genes outside of clusters.

The results can also be explored for each comparison between the infection stage.

Hide

```
as.data.frame(table(significant$Gene_kind,
                    significant$Comparison, significant$Sample)) %>%
  pivot_wider(names_from = Var1, values_from = Freq) %>%
  dplyr::select(-Var3) %>%
  kbl() %>%
  kable_styling(bootstrap_options = "striped", full_width = F, position = "left") %>%
  pack_rows("Zt05", 1, 3)%>%
  pack_rows("Zt09", 4, 6)%>%
  pack_rows("Zt10", 7, 9)
```

Var2	biosynthetic	biosynthetic-additional	other	transport
**Zt05**				
A_B	6	10	0	2
B_C	6	12	1	3
C_D	3	4	0	0
**Zt09**				
A_B	10	13	0	4
B_C	8	16	0	10
C_D	8	13	0	3
**Zt10**				
A_B	2	5	0	1
B_C	9	16	1	4
C_D	2	1	0	1

Hide

```
temp =as.data.frame(table(significant$Gene_kind,
                    significant$Comparison, significant$Sample)) %>%
  pivot_wider(names_from = Var1, values_from = Freq)

temp[order(temp$Var2),] %>%
  dplyr::select(-Var2) %>%
  kbl() %>%
  kable_styling(bootstrap_options = "striped", full_width = F, position = "left") %>%
  pack_rows("A to B", 1, 3)%>%
  pack_rows("B to C", 4, 6)%>%
  pack_rows("C to D", 7, 9)
```

Var3	biosynthetic	biosynthetic-additional	other	transport
**A to B**				
Zt05	6	10	0	2
Zt09	10	13	0	4
Zt10	2	5	0	1
**B to C**				
Zt05	6	12	1	3

Var3	biosynthetic	biosynthetic-additional	other	transport
Zt09	8	16	0	10
Zt10	9	16	1	4
**C to D**				
Zt05	3	4	0	0
Zt09	8	13	0	3
Zt10	2	1	0	1

I now want to be able to visualize the fact that differences in expression are significant or not. Significant changes will be represented by a full line whereas non-significant changes will be dotted lines.

Hide

```
#Assembling data and transforming the frame to get the right format for geom_segments
for_plot_significant = In_planta_diff_results %>%
  dplyr::select(Gene, Diff, Comparison, Sample, Cluster_Amine,
                Cluster_Function, Biosynthetic_gene_nb, BGF) %>%
  filter(Diff == "Significant") %>%
  unique() %>%
  left_join(., average_TPM) %>%
  mutate(Condition_simple = gsub("in_planta_", "", Condition)) %>%
  dplyr::select(Gene, Sample, Comparison, Condition_simple, av_TPM,
                Cluster_Function, BGF, Cluster_Amine_underscore,
                Gene_kind,  Orthogroup, Biosynthetic_gene_nb) %>%
  separate(Comparison, into = c("Condition1", "Condition2"), sep = "_", remove = FALSE) %>%
  mutate(temp = ifelse(Condition_simple == Condition1 | Condition_simple == Condition2, "Yeah", "Nope")) %>%
  mutate(Position = ifelse(Condition_simple == Condition1, "Start", "End")) %>%
  filter(temp == "Yeah") %>%
  dplyr::select(-temp, -Condition_simple) %>%
  pivot_wider(names_from = Position, values_from = av_TPM)


#Once I have the data frame, I subset the bits that I want
temp = for_plot_significant %>%
  filter(Gene_kind == "biosynthetic") %>%
  filter(!is.na(Cluster_Function))

#... and then I can add the lines to the plot as created before!
plot_TPM_knownBGC +
  geom_segment(data = temp,
               aes(x = Condition1, xend = Condition2,
                                                 y = Start, yend = End))
```

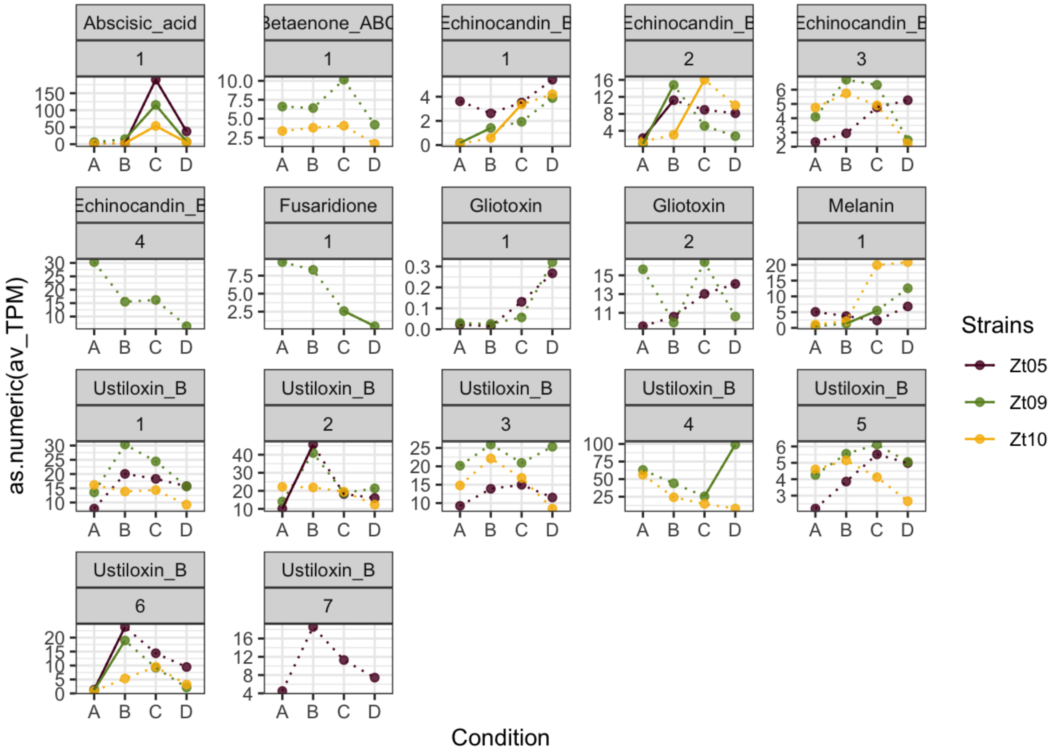

From this it looks like the clearest pattern here is with the abscisic acid. Let’s look at all the genes constituting the cluster.

Hide

```
cluster_functions = unique(known_BGC_core$Cluster_Function)


plot_per_BGC = list()
for (cl_func in cluster_functions){

plot_per_BGC[[cl_func]] =
  known_BGC %>%
  filter(Cluster_Function == cl_func) %>%
  left_join(., average_TPM, by = c("Sample", "Cluster_Amine")) %>%
  filter(Condition !=  "in_vitro_kmt6") %>%
  filter(Condition !=  "in_vitro_wild") %>%
  mutate(Condition = gsub("in_planta_", "", Condition)) %>%
    ggplot(aes(x = Condition, y = as.numeric(av_TPM), color = Sample)) +
    geom_point(alpha = 0.8) +
    geom_line(aes(group = Sample), linetype ="dotted") +
    facet_wrap(vars(Orthogroup), scales = "free") +
    theme_bw() +
    scale_color_strains +
    labs(title = "Transcriptomic profiles of orthologs",
         subtitle = paste0("Found in the cluster predicted to produce ", cl_func))
}


#Adding segments to the plots for each cluster
plot_per_BGC[[cluster_functions[[1]]]]+
  geom_segment(data = for_plot_significant %>% filter(Cluster_Function == cluster_functions[[1]]),
               aes(x = Condition1, xend = Condition2, y = Start, yend = End))
```

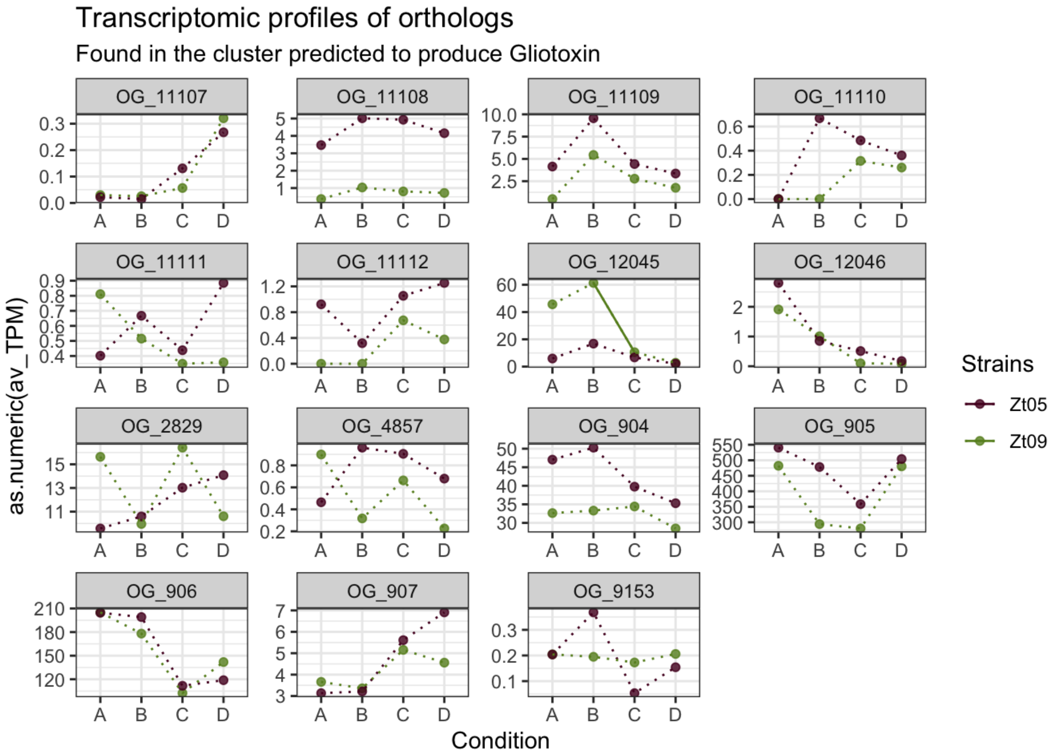

Hide

```
plot_per_BGC[[cluster_functions[[3]]]] +
  geom_segment(data = for_plot_significant %>% filter(Cluster_Function == cluster_functions[[3]]),
               aes(x = Condition1, xend = Condition2, y = Start, yend = End))
```

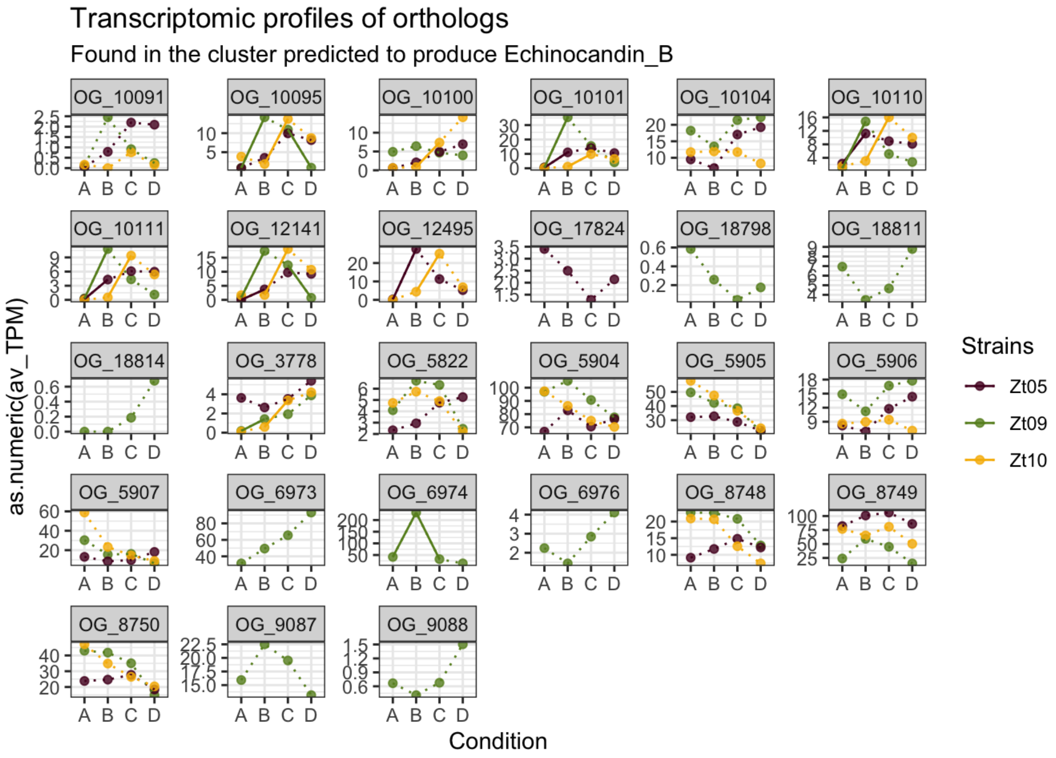

Hide

```
plot_per_BGC[[cluster_functions[[4]]]] +
  geom_segment(data = for_plot_significant %>% filter(Cluster_Function == cluster_functions[[4]]),
               aes(x = Condition1, xend = Condition2, y = Start, yend = End))
```

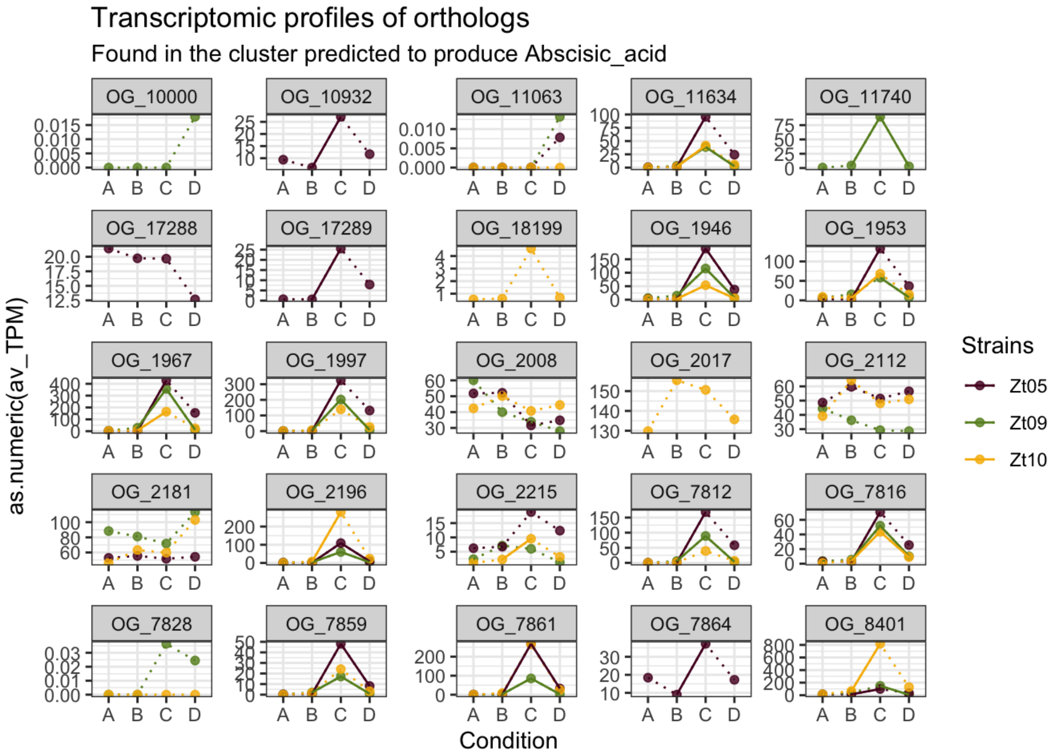

Hide

```
## The second cluster is part of the main figure, so I save the plot separately for this one
p = plot_per_BGC[[cluster_functions[[2]]]] +
  geom_segment(data = for_plot_significant %>% filter(Cluster_Function == cluster_functions[[2]]),
               aes(x = Condition1, xend = Condition2, y = Start, yend = End))
p
```

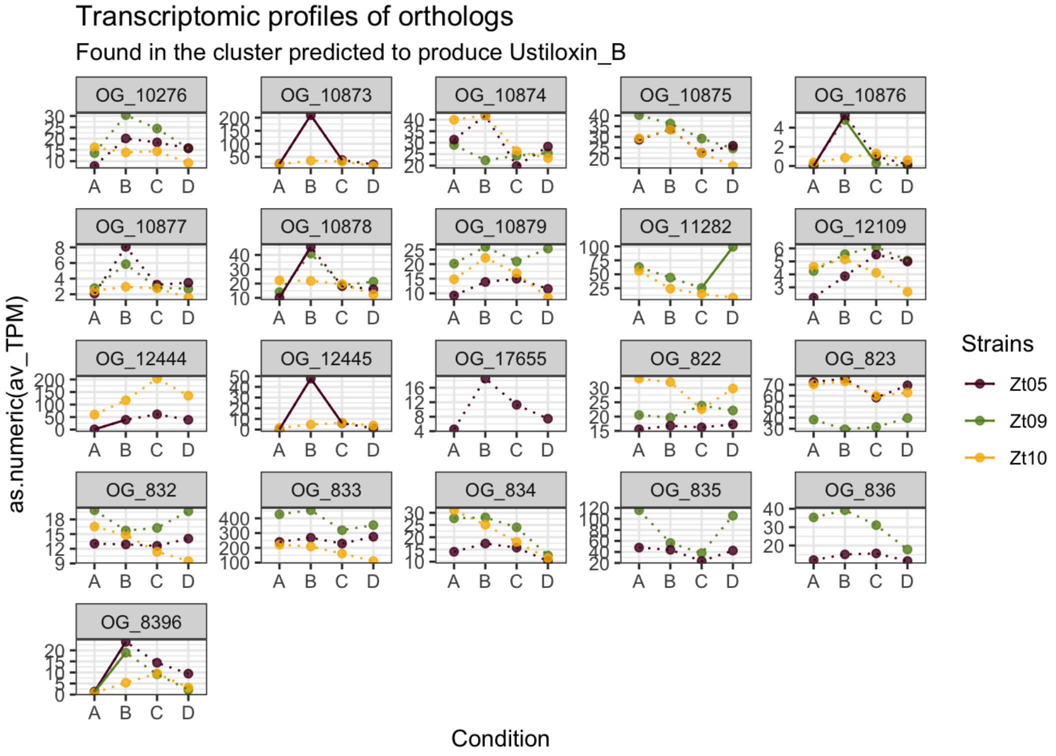

Hide

```
ggsave(paste0(work_dir, "Expression_AA.pdf"), p, width = 16, height = 15, units = "cm")
```

And now for all core biosynthetic genes from all clusters:

Hide

```
#Transforming the data in the right format
temp = average_TPM %>%
  filter(Gene_kind == "biosynthetic") %>%
  ungroup() %>%
  dplyr::select(BGF, Orthogroup, Condition, Sample, av_TPM) %>%
  filter(Condition !=  "in_vitro_kmt6") %>%
  filter(Condition !=  "in_vitro_wild") %>%
  mutate(Condition = gsub("in_planta_", "", Condition)) %>%
  unite(BGF, Orthogroup, col = Ortho, sep = ":", remove = F)

orthologs_biosynthetic = sort(unique(temp$Ortho))

temp2 = for_plot_significant %>%
  unite(BGF, Orthogroup, col = Ortho, sep = ":", remove = F)


#Generating the plots
p1 = temp %>%
    filter(Ortho %in% orthologs_biosynthetic[1:16]) %>%
    ggplot(aes(x = Condition, y = as.numeric(av_TPM), color = Sample)) +
    geom_point() +
    geom_line(aes(group = Sample), linetype = "dotted") +
    facet_wrap(vars(Ortho), scales = "free") +
    theme_bw() +
    scale_color_strains +
  geom_segment(data = temp2 %>% filter(Ortho %in% orthologs_biosynthetic[1:16]),
               aes(x = Condition1, xend = Condition2, y = Start, yend = End)) +
  labs(x = "Infection stage", y = "TPM (averaged over replicates)")

p2 = temp %>%
    filter(Ortho %in% orthologs_biosynthetic[17:32]) %>%
    ggplot(aes(x = Condition, y = as.numeric(av_TPM), color = Sample)) +
    geom_point() +
    geom_line(aes(group = Sample), linetype = "dotted") +
    facet_wrap(vars(Ortho), scales = "free") +
    theme_bw() +
    scale_color_strains +
  geom_segment(data = temp2 %>% filter(Ortho %in% orthologs_biosynthetic[17:32]),
               aes(x = Condition1, xend = Condition2, y = Start, yend = End))+
  labs(x = "Infection stage", y = "TPM (averaged over replicates)")

p3 = temp %>%
    filter(Ortho %in% orthologs_biosynthetic[33:length(orthologs_biosynthetic)]) %>%
    ggplot(aes(x = Condition, y = as.numeric(av_TPM), color = Sample)) +
    geom_point() +
    geom_line(aes(group = Sample), linetype = "dotted") +
    facet_wrap(vars(Ortho), scales = "free") +
    theme_bw() +
    scale_color_strains +
  geom_segment(data = temp2 %>% filter(Ortho %in% orthologs_biosynthetic[33:length(orthologs_biosynthetic)]),
               aes(x = Condition1, xend = Condition2, y = Start, yend = End))+
  labs(x = "Infection stage", y = "TPM (averaged over replicates)")


#Show the plot in the Rmd hmtl
p1
```

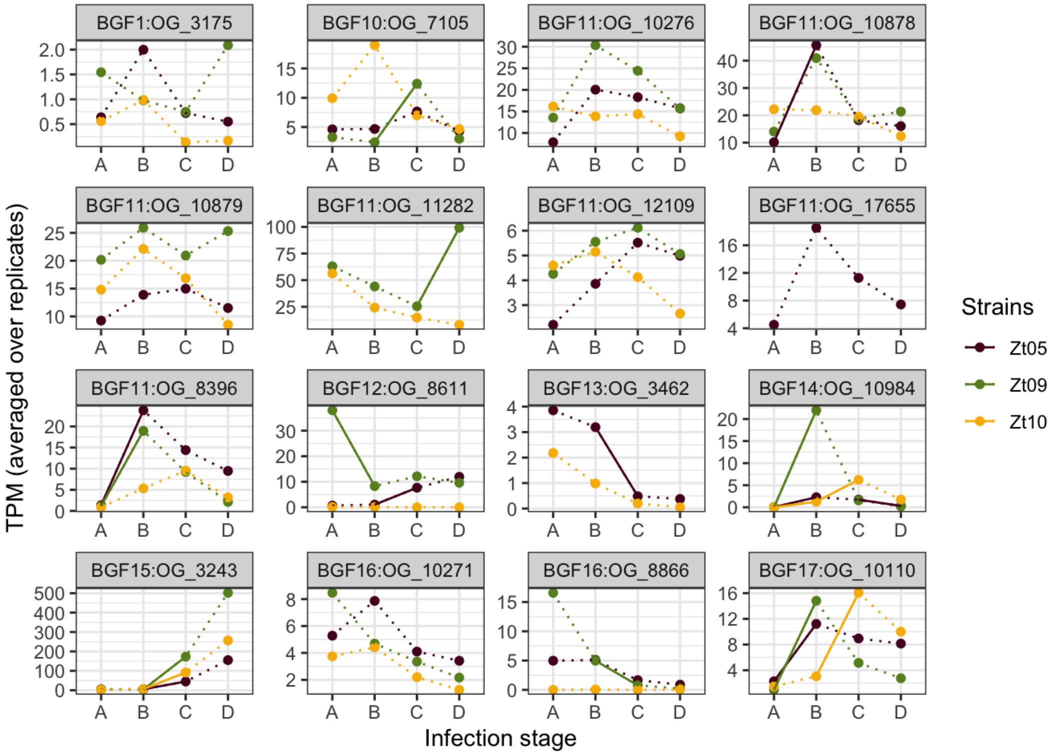

Hide

p2

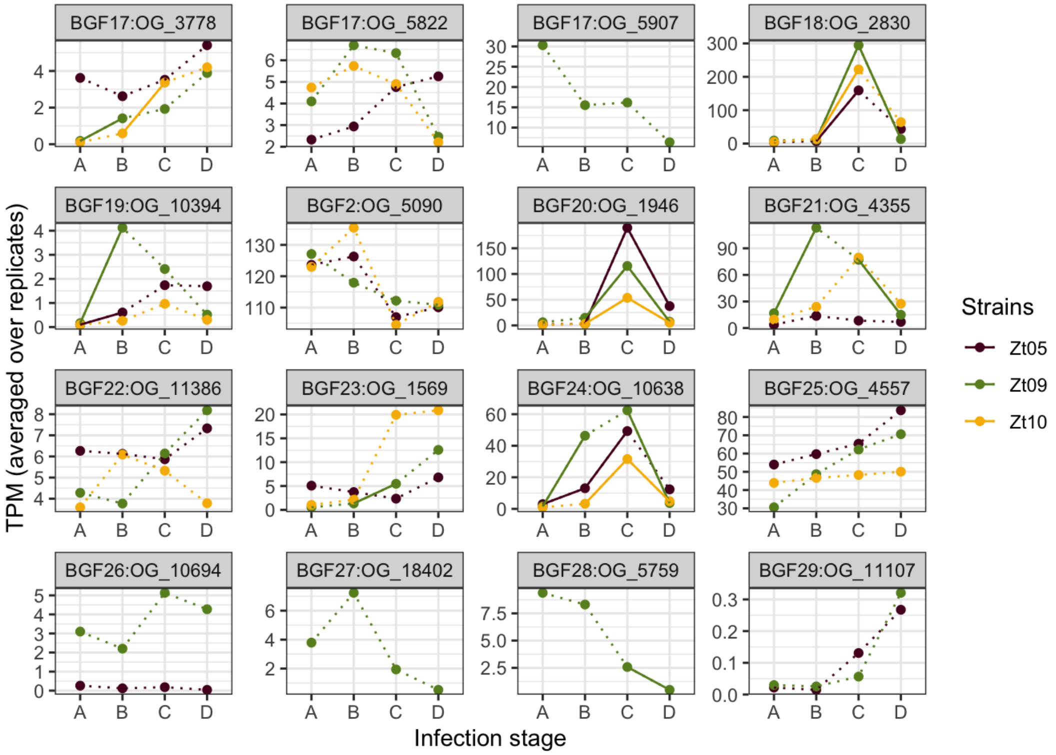

Hide

p3

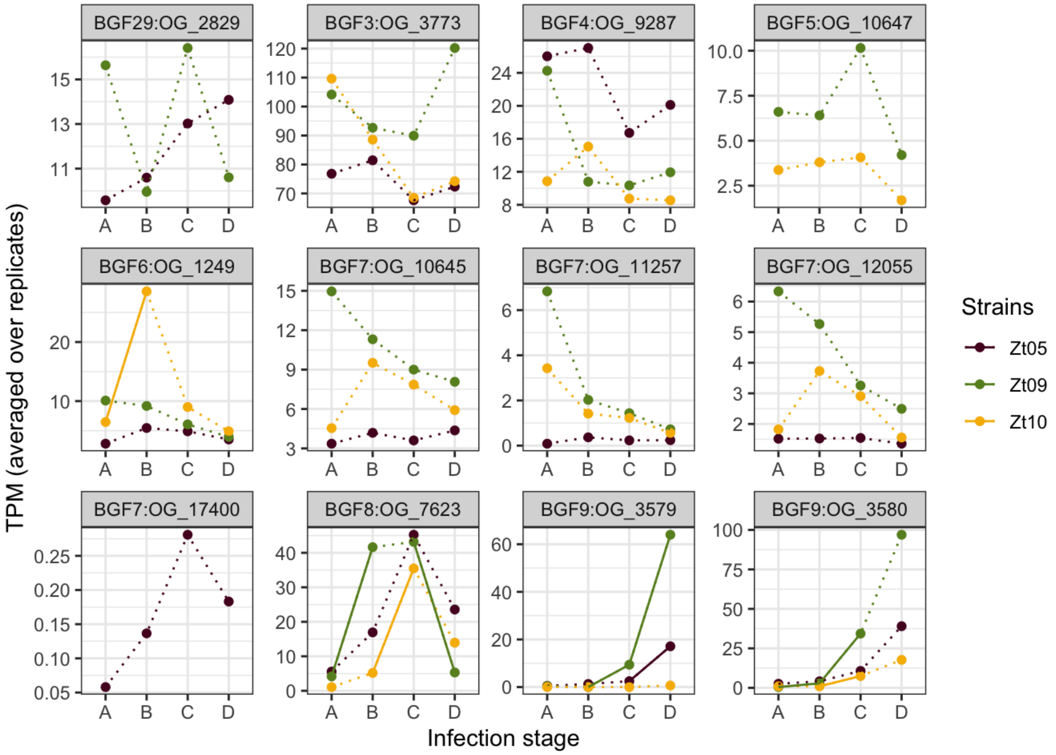

Hide

```
#Save the plots for the supplementary figure

pdf(paste0(to_publish_dir,"Fig_S1.pdf"), 7, 5)
p1
p2
p3
dev.off()
```

```
## quartz_off_screen
##                 2
```

I find it interesting to plot each stage as there does not seem to be a clear cut bio vs necro phases. For instance, the orthogroups 2830, 10638 and 1946 all show a peak of expression in stage C.

Synteny plots

I will first make a synteny plot for the melanin cluster which is the only one with a 100% similarity score in antiSMASH analysis

Hide

```
temp = gene_details %>%
    filter(Cluster_Function == "Melanin") %>%
    mutate(direction = ifelse(Strand == "-", FALSE, TRUE))

ggplot(temp, aes(xmin = Start, xmax = End, y = Sample, fill = Gene_kind,
                 forward = direction, label = Orthogroup)) +
  geom_gene_arrow() +
  geom_gene_label() +
  geom_text(aes(x = Start , y = Sample), size = 2, nudge_y = 0.15) +
  facet_wrap(~ Sample, scales = "free", ncol = 1) +
  theme_genes() +
  theme(legend.position = "bottom",
        axis.text.x = element_text(size=6)) +
  labs(ylab = "", xlab = "Coordinates on contig")
```

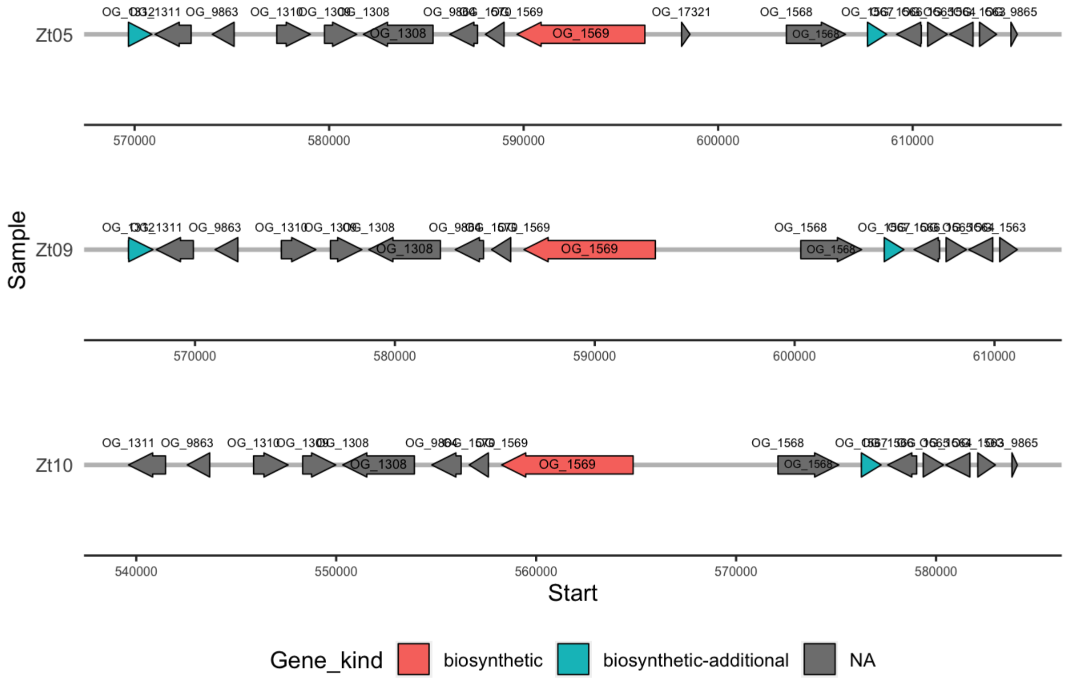

And then, do the same plot but for the abscisic acid this time.

Hide

```
temp =gene_details %>%
    filter(Cluster_Function == "Abscisic_acid")  %>%
    mutate(direction = ifelse(Strand == "-", FALSE, TRUE)) %>%
   mutate(Start = ifelse(Sample == "Zt10", -Start, Start))%>%
   mutate(End = ifelse(Sample == "Zt10", -End, End))

temp2 = temp %>% filter(Sample == "Zt05") %>% dplyr::select(Gene, Orthogroup, Start)

p = ggplot(temp, aes(xmin = Start, xmax = End, y = Sample, fill = Gene_kind,
                 forward = direction, label = Orthogroup)) +
  geom_gene_arrow() +
  geom_gene_label() +
  geom_text(aes(x = Start , y = Sample), size = 2, nudge_y = 0.15) +
  facet_wrap(~ Sample, scales = "free", ncol = 1) +
  theme_genes() +
  theme(legend.position = "bottom",
        axis.text.x = element_text(size=6)) +
  labs(ylab = "", xlab = "Coordinates on contig")

p
```

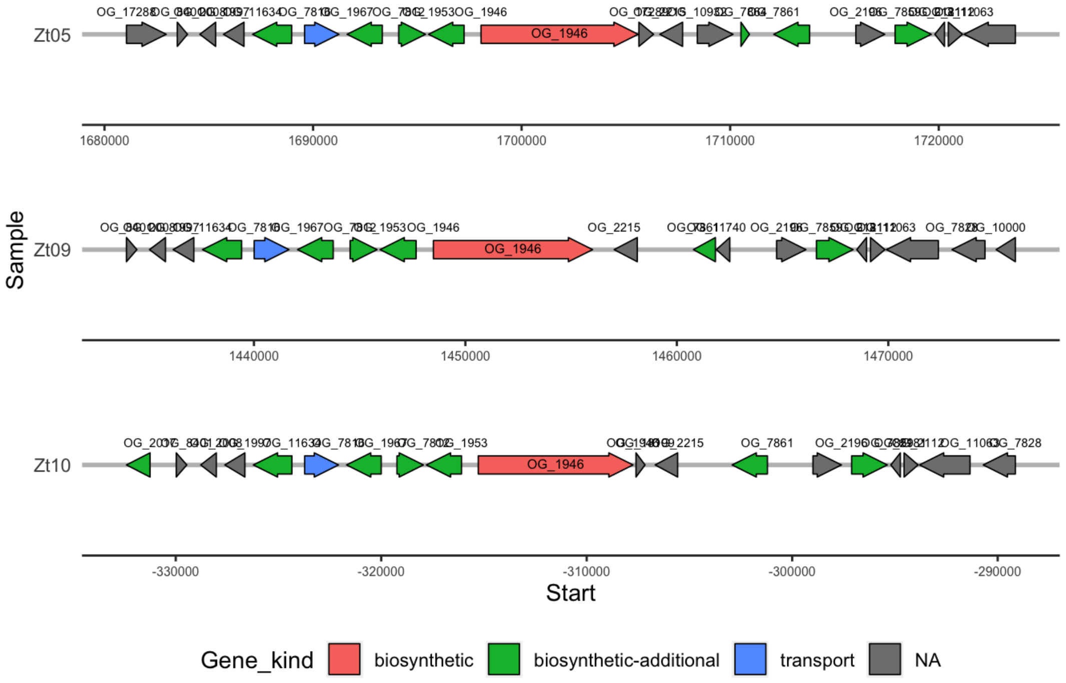


```
ggsave(paste0(work_dir, "Synteny_AA.pdf"), p)
```

It looks like the main biosynthetic gene from the **melanin** cluster is more expressed in necrotrophic phase in Zt10, specifically. The biosynthetic gene from what is predicted as a possible **abscisic acid** cluster has an increased expression in stage C. From the wikipedia page (https://en.wikipedia.org/wiki/Abscisic_acid#:~:text=Abscisic%20acid%20(ABA)%20is%20a,organ%20size%20and%20stomatal%20closure.) of this compound it would seem that this is mainly a plant hormone but that some fungi are known to produce it. It is hypothesized that this play a role in **suppressing the plant immune response**. So it might be good to investigate this a bit more and see if we can get a clearer idea of whether this prediction can be strengthened.

In vitro: kmt6 vs wild type

```
#Reading the data
sampleFiles_invitro <- grep(suffix_RC,
                    list.files(paste0(RC_dir,"In_vitro")),
                    value=TRUE)
sampleCondition_invitro <- c("in_vitro_wild", "in_vitro_wild", "in_vitro_kmt6", "in_vitro_kmt6" )
sampleTable_invitro <- data.frame(sampleName = sampleFiles_invitro ,
                          fileName = sampleFiles_invitro ,
                          condition = sampleCondition_invitro)
sampleTable_invitro$condition  <- factor(sampleTable_invitro$condition )

#Read the data into DESeq2 format
dds_invitro  <- DESeqDataSetFromHTSeqCount(sampleTable = sampleTable_invitro ,
                                       directory = paste0(RC_dir,"In_vitro"),
                                       design= ~ condition)
dds_invitro
```

```
## class: DESeqDataSet
## dim: 11810 4
## metadata(1): version
## assays(1): counts
## rownames(11810): Zt09_chr_10_00001 Zt09_chr_10_00002 ...
##   Zt09_chr_9_00628 Zt09_chr_9_00629
## rowData names(0):
## colnames(4): S0241_Zt09_1_A1_read_counts_woTEgenes.txt
##   S0242_Zt09_2_A2_read_counts_woTEgenes.txt
##   S0245_kmt6_1_A5_read_counts_woTEgenes.txt
##   S0246_kmt6_2_A6_read_counts_woTEgenes.txt
## colData names(1): condition
```

Hide

```
#Pre-filtering of low values in_vitro
keep <- rowSums(counts(dds_invitro)) >= 5
dds_invitro <- dds_invitro[keep,]

dds_invitro <- DESeq(dds_invitro)

resultsNames(dds_invitro)
```

```
## [1] "Intercept"
## [2] "condition_in_vitro_wild_vs_in_vitro_kmt6"
```

Hide

```
reswild_vs_kmt6 <- results(dds_invitro, name="condition_in_vitro_wild_vs_in_vitro_kmt6", test="Wald")

as_tibble(reswild_vs_kmt6, rownames = "Gene")  %>%
  left_join(., gene_details, by = "Gene") %>%
  dplyr::select(Gene, Cluster_Amine, Cluster_Function, BGF, Gene_kind,
              baseMean, log2FoldChange, lfcSE, stat, pvalue, padj) %>%
  rename(Cluster_Amine = "BGC") %>%
  write_tsv(paste0(to_publish_dir, "Differential_expression_in_vitro.txt"))


#Make pretty plots to compare repeats and conditions
vsd <- vst(dds_invitro, blind=FALSE)

sampleDists <- dist(t(assay(vsd)))
sampleDistMatrix <- as.matrix(sampleDists)
rownames(sampleDistMatrix) <- paste(vsd$condition, vsd$type, sep="-")
colnames(sampleDistMatrix) <- NULL
colors <- colorRampPalette( rev(brewer.pal(9, "Blues")) )(255)
pheatmap(sampleDistMatrix,
         clustering_distance_rows=sampleDists,
         clustering_distance_cols=sampleDists,
         col=colors)
```

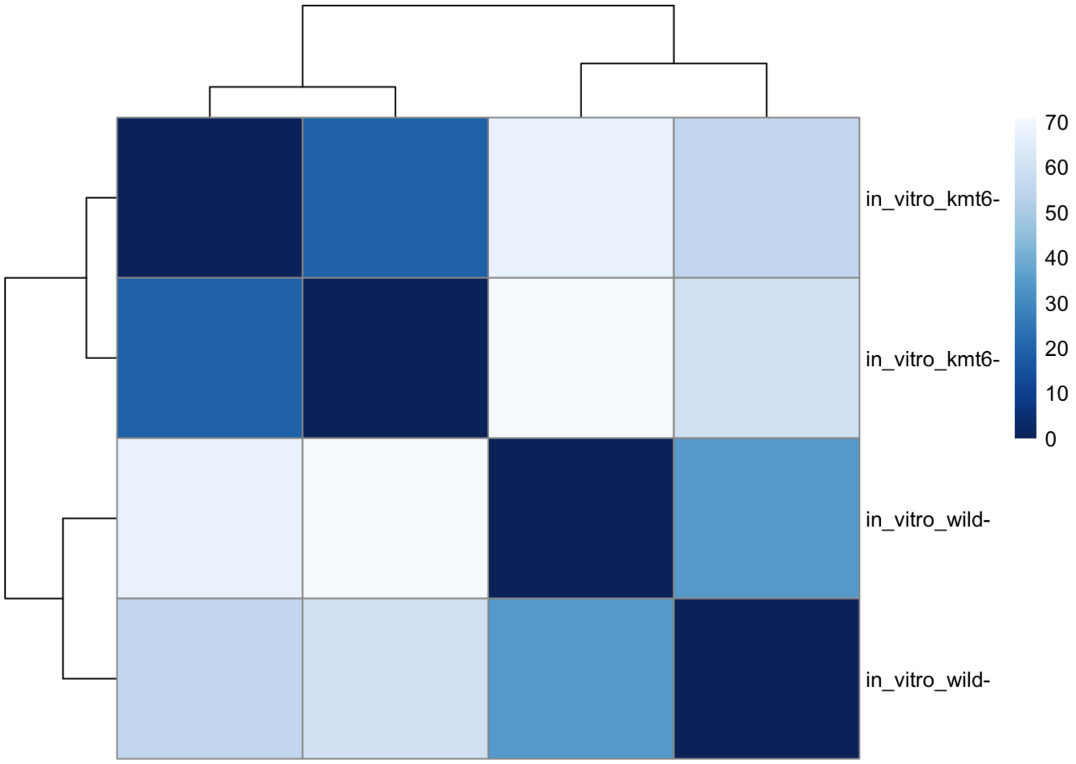

Hide

```
pcaData <- plotPCA(vsd, intgroup="condition", returnData=TRUE)
percentVar <- round(100 * attr(pcaData, "percentVar"))
ggplot(pcaData, aes(PC1, PC2, color=condition)) +
  geom_point(size=3) +
  xlab(paste0("PC1: ",percentVar[1],"% variance")) +
  ylab(paste0("PC2: ",percentVar[2],"% variance")) +
  coord_fixed() + theme_bw()
```

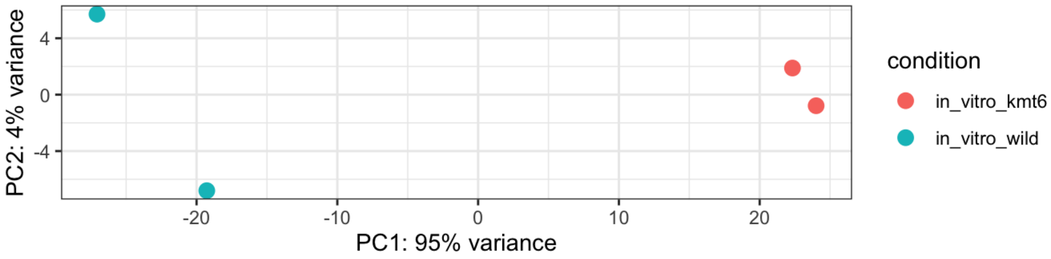

Unsurprisingly, there is a very clear cut difference between the expression profile of the kmt6 mutant and the wild. This difference is overwhelmingly higher than the one between repeats of the same strain.

Now, let’s look for differentially expressed genes that are relevant to our topic. I used threshold which are similar to Mareike’s since I assumed she knew her dataset well.

Hide

```
#Analysis
temp = as_tibble(reswild_vs_kmt6, rownames = "Gene")  %>%
  mutate(Diff = ifelse(padj <= 0.001,
                       ifelse(abs(log2FoldChange) > 2, "Significant", "Not_significant"),
                       "Not_significant")) %>%
  left_join(., gene_details, by = "Gene") %>%
  mutate(BGC = ifelse(is.na(Cluster_Amine), "Not_BGC", "BGC"))

#table(temp$Diff)
as.data.frame(table(temp$Diff, temp$BGC)) %>%
  pivot_wider(names_from = Var2, values_from = Freq) %>%
  kbl() %>%
  kable_styling(bootstrap_options = "striped", full_width = F, position = "left")
```

Var1	BGC	Not_BGC
Not_significant	312	9857
Significant	6	375

Hide

```
as.data.frame(table(temp$Diff, temp$Gene_kind)) %>%
  pivot_wider(names_from = Var2, values_from = Freq) %>%
  kbl() %>%
  kable_styling(bootstrap_options = "striped", full_width = F, position = "left")
```

Var1	biosynthetic	biosynthetic-additional	other	regulatory	transport
Not_significant	37	47	5	1	23
Significant	0	1	1	0	1

Hide

```
plot_2 = as.data.frame(table(temp$Diff, temp$BGC))   %>%
  group_by(Var2) %>%
  mutate(perc = Freq/sum(Freq))  %>%
  ggplot(aes(Var2, perc, fill = Var1)) +
  geom_bar(stat = "identity")
```

As previously found by Mareike, there are very few genes which show a change in expression in the kmt6 mutant *in vitro*. There are even fewer differentially expressed genes with a known function in one of the BGC: indeed, none of the main biosynthetic genes are differentially expressed and only one each of a predicted transporter and biosyntethic “additional” gene.
